# Evaluation of *In Vitro* Cross-Reactivity to Avian H5N1 and Pandemic H1N1 2009 Influenza Following Prime Boost Regimens of Seasonal Influenza Vaccination in Healthy Human Subjects: A Randomised Trial

**DOI:** 10.1371/journal.pone.0059674

**Published:** 2013-03-26

**Authors:** Delia Bethell, David Saunders, Anan Jongkaewwattana, Jarin Kramyu, Arunee Thitithayanont, Suwimon Wiboon-ut, Kosol Yongvanitchit, Amporn Limsalakpetch, Utaiwan Kum-Arb, Nichapat Uthaimongkol, Jean Michel Garcia, Ans E. Timmermans, Malik Peiris, Stephen Thomas, Anneke Engering, Richard G. Jarman, Duangrat Mongkolsirichaikul, Carl Mason, Nuanpan Khemnu, Stuart D. Tyner, Mark M. Fukuda, Douglas S. Walsh, Sathit Pichyangkul

**Affiliations:** 1 Department of Immunology and Medicine, Armed Forces Research Institute of Medical Sciences, Bangkok, Thailand; 2 Virology and Cell Technology Laboratory, National Center for Genetic Engineering and Biotechnology, Bangkok, Thailand; 3 Faculty of Science, Mahidol University, Bangkok, Thailand; 4 University of Hong Kong-Pasteur Research Center, Hong Kong Special Administrative Region, China; 5 Department of Virology, Armed Forces Research Institute of Medical Sciences, Bangkok, Thailand; 6 Department of Enteric Diseases, Armed Forces Research Institute of Medical Sciences, Bangkok, Thailand; University of Rochester Medical Center, United States of America

## Abstract

**Introduction:**

Recent studies have demonstrated that inactivated seasonal influenza vaccines (IIV) may elicit production of heterosubtypic antibodies, which can neutralize avian H5N1 virus in a small proportion of subjects. We hypothesized that prime boost regimens of live and inactivated trivalent seasonal influenza vaccines (LAIV and IIV) would enhance production of heterosubtypic immunity and provide evidence of cross-protection against other influenza viruses.

**Methods:**

In an open-label study, 26 adult volunteers were randomized to receive one of four vaccine regimens containing two doses of 2009-10 seasonal influenza vaccines administered 8 (±1) weeks apart: 2 doses of LAIV; 2 doses of IIV; LAIV then IIV; IIV then LAIV. Humoral immunity assays for avian H5N1, 2009 pandemic H1N1 (pH1N1), and seasonal vaccine strains were performed on blood collected pre-vaccine and 2 and 4 weeks later. The percentage of cytokine-producing T-cells was compared with baseline 14 days after each dose.

**Results:**

Subjects receiving IIV had prompt serological responses to vaccine strains. Two subjects receiving heterologous prime boost regimens had enhanced haemagglutination inhibition (HI) and neutralization (NT) titres against pH1N1, and one subject against avian H5N1; all three had pre-existing cross-reactive antibodies detected at baseline. Significantly elevated titres to H5N1 and pH1N1 by neuraminidase inhibition (NI) assay were observed following LAIV-IIV administration. Both vaccines elicited cross-reactive CD4+ T-cell responses to nucleoprotein of avian H5N1 and pH1N1. All regimens were safe and well tolerated.

**Conclusion:**

Neither homologous nor heterologous prime boost immunization enhanced serum HI and NT titres to 2009 pH1N1 or avian H5N1 compared to single dose vaccine. However heterologous prime-boost vaccination did lead to *in vitro* evidence of cross-reactivity by NI; the significance of this finding is unclear. These data support the strategy of administering single dose trivalent seasonal influenza vaccine at the outset of an influenza pandemic while a specific vaccine is being developed.

**Trial Registration:**

ClinicalTrials.gov NCT01044095

## Introduction

The threat of pandemic influenza remains a major public health concern. In recent years, several avian viruses have crossed the species barrier and directly infected humans, presenting a possible pandemic threat. One of these is avian influenza H5N1 virus, which has a mortality rate of more than 50% in the 600 laboratory-confirmed human cases reported by WHO since 2003 [1]. Current seasonal trivalent influenza vaccines rely on predicted antigens based on the previous season's circulating viruses, and do not allow for the sudden antigenic shift that leads to a pandemic; moreover development of a specific vaccine against a new pandemic virus takes time. Efforts are now focussed on the search for a universal influenza vaccine that can confer broad and long-lasting protection to all types of influenza.

Live, attenuated influenza vaccine (LAIV) is an intranasally administered vaccine, designed to induce an immune response resembling infection with wild-type influenza without causing disease [2]. Compared to conventional intramuscular inactivated vaccines (IIV), LAIV is believed to induce mucosal antibody responses and cellular immunity [3]. Moreover, LAIV can induce responses to antigenically mismatched influenza A strains [4]. It has been well documented that heterologous prime boost vaccination elicits high-magnitude, broad-based and long-lasting immunity in several different animal and disease models [5] [6] [7]. Recent work in mice, ferrets and monkeys demonstrated that a prime boost strategy of a DNA vaccine followed by seasonal IIV conferred protection against a range of influenza viruses by inducing broadly neutralizing antibodies against the stem cell region of the haemagglutinin (HA) glycoprotein [8].

We describe here a pilot feasibility study designed to test the hypothesis that heterologous prime-boost immunization of healthy humans with seasonal trivalent LAIV and IIV would induce evidence of *in vitro* cross-protection against non-vaccine influenza viruses such as avian H5N1 and pandemic 2009 H1N1.

## Materials and Methods

### Ethics Statement

The study was approved by the Walter Reed Army Institute of Research (WRAIR) IRB (FWA00000015) and governed by ICH GCP guidelines.

### Design

This was a randomized, open-label, pilot feasibility study of four two-dose vaccine regimens using two commercially available trivalent seasonal influenza vaccines to compare immune responses and *in vitro* cross-reactivity against avian H5N1 and pandemic 2009 H1N1 viruses. The protocol for this trial and supporting CONSORT checklist are available as supporting information; see Checklist S1 and Protocol S1.

### Subjects

26 healthy U.S. citizens living in Bangkok, Thailand, aged 18–49 years who had not received influenza vaccination (either seasonal or 2009 pandemic H1N1) within the preceding 6 months were recruited into the study if they tested negative for HIV and had a normal complete blood count at screening. All subjects gave written informed consent prior to study participation.

### Location

All vaccine doses were administered at the US Embassy Medical Unit, Bangkok, Thailand. Other clinical activities were conducted either at the US Embassy Medical Unit, Bangkok and/or the Department of Immunology and Medicine, AFRIMS, Bangkok.

### Clinical Methods

Vaccine administration. The vaccines used were FluMist® intranasal live, attenuated influenza virus (LAIV) vaccine (dose 0.1 mL per nostril, supplied by MedImmune, Gaithersburg, MD); and Fluzone® inactivated influenza virus (IIV) vaccine (dose 0.5 mL intramuscularly, purchased in Thailand from Sanofi-Pasteur). Both vaccines contained the three strains for the 2009/10 northern hemisphere season: A/South Dakota/6/2007 (H1N1) (an A/Brisbane/59/2007-like), A/Uruguay/716/2007 (H3N2) (an A/Brisbane/10/2007-like), and B/Brisbane/60/2008. Enrolled subjects were allocated consecutively numbered sealed envelopes containing one of four vaccine regimens: homologous prime boost regimen 1 (Group 1): LAIV two doses separated by 8 weeks (±7 days), n = 5; homologous prime boost regimen 2 (Group 2): IIV two doses separated by 8 weeks (±7 days), n = 5; heterologous prime boost regimen 1 (Group 3), LAIV single dose, followed by IIV single dose 8 weeks (±7 days) later, n = 8; heterologous prime boost regimen 2 (Group 4): IIV single dose followed by LAIV single dose 8 weeks (±7 days) later, n = 8. The randomization code was computer-generated and the key maintained by a research nurse independent from the study team.

#### Sample collection

Antibody and cellular responses were measured in blood and following nasal irrigation with 20 ml warmed 0.9% saline two and four weeks after each vaccine dose and compared with baseline values. Blood for serum separation was collected in SST (Serum Separation Tube with clot activator and gel). For peripheral blood mononuclear cells, blood was collected in heparinized tubes and cells separated by histoplaque using Leucosep tubes. Serum samples and peripheral blood mononuclear cells were stored in liquid nitrogen until use. All laboratory staff were blinded as to allocation of vaccine regimens until the study database was locked.

#### Adverse event (AE) reporting

Because the current recommendation for healthy adults is to receive a single dose of seasonal trivalent influenza vaccine, and because of a lack of published data on the safety of two doses in adults, detailed safety data was collected for the duration of this study. All subjects were asked to complete a Symptom Diary daily for 14 days following each dose of vaccine. All AEs that were reported and all concomitant medication used, whether or not attributed or related to vaccine administration, were recorded from the time of first phlebotomy until the end of study participation. AEs were documented individually, not by syndrome, and classified according to MedDRA® (the Medical Dictionary for Regulatory Activities), which is international medical terminology developed under the auspices of the International Conference on Harmonization of Technical Requirements for Registration of Pharmaceuticals for Human Use. Each AE was described by its duration, an assessment of causality (vaccine, coexisting disease, due to concomitant medication, or others), relationship to vaccine (not related, unlikely, possibly, probably, definitely), and whether specific therapy was required. The subject and investigator made an assessment of severity for each reported AE as follows: mild (self-limiting or minor symptoms that did not affect activities of daily living and did not require treatment); moderate (symptoms that required treatment in order to carry out activities of daily living); and severe (symptoms preventing activities of daily living and requiring out-patient treatment).

### Laboratory Methods

#### Haemagglutination inhibition (HI) assay

Sera were treated with receptor-destroying enzyme and subsequently heat-inactivated. The haemagglutination inhibition assay was performed by WHO standard methods using 8 HA units of influenza virus. Guinea pig red blood cells were used for seasonal influenza viruses and 2009 pH1N1 and goose red blood cells were used for avian influenza H5N1. Samples were tested in serial 2-fold dilutions by starting at 1∶10 dilution. The antibody titres were defined as the reciprocal of the highest dilution of sera samples that completely inhibits haemagglutination. Titres that were lower than the detection limit were assigned a value of 5 for analysis of geometric mean titre (GMT).

#### Neutralization (NT) assays

*Influenza A (H5) pseudotyped lentiviral particle (H5pp)-based serological assay.* H5 haemagglutinin (A/Cambodia/408008/05; clade 1) pseudotyped lentiviral particles were obtained from HKU-Pasteur Research Center. The assay was conducted as previously described [9]. Samples were tested in serial 2-fold dilutions by starting at 1∶20 dilution. The neutralization titre was defined as the reciprocal of the highest dilution of sera samples that inhibited 50% infection. Samples that tested negative at 1∶10 were assigned a titre of 1∶5 for analysis of geometric mean titre (GMT).

#### Microneutralization assay for 2009 pH1N1

To detect antibodies that could inhibit infection of cells with influenza virus, microneutralization assays were performed using Madin-Darby canine kidney (MDCK) cells. Samples were heat-inactivated (30 min at 56°C) and serial dilutions pre-incubated with 2009 H1N1 (A/California/04/2009:100 TCID_50_) in 96-well plates. After 1–2 h incubation at 37°C in a 5% CO2, the mixtures were added to a pre-formed monolayer of MDCK cells and the plates were incubated for another 18 hours. MDCK monolayers were then washed with PBS and fixed in cold 80% acetone for 10 min. The presence of viral protein was detected by ELISA using a monoclonal antibody to the influenza A nucleoprotein (NP). The second antibody conjugated with peroxidase was added and incubated for another 1 h. Plates were washed, and specific enzyme substrate added. The reactions were stopped with 1 N sulphuric acid. The absorbance was measured at 490 nm. The average A490 was determined for quadruplicate wells of virus-infected (VC) and –uninfected (CC) control wells, and a neutralizing endpoint determined by using a 50% specific signal calculation. The endpoint titre was expressed as the reciprocal of the highest dilution of serum with A490 value less than X, where X = [(average A490 of VC wells) - (average A490 of CC wells)]/2+(average A490 of CC wells). Sera, which tested negative at a dilution of 1∶20, were assigned a titre of 1∶10 for analysis of GMT.

#### Antibody staining against H5N1 matrix 2 protein (M2e) expressed cell line intensity

HEK 293 stably expressing H5N1 M2e (A/Vietnam/1203/04) on the cell surface was provided by Dr. M. Moyle (Theraclone Science, WA). Cells were stained with a 1∶10 dilution of sera samples and then detected by florescent dye conjugated anti-human antibodies [10]. Mean fluorescence intensity of stained cells was analyzed by flow cytometry.

#### Neuraminidase inhibition (NI) assay

Sera were assayed for antibodies against neuraminidase by a standard colorimetric neuraminidase inhibition method [11]. Samples were tested in serial 2-fold dilutions by starting at 1∶160. The neutralization titre was defined as the reciprocal of the highest dilution of sera samples that inhibited 50% of neuraminidase activity. Titres that were lower than the detection limit were assigned a value of 1∶80 for analysis of GMT.

#### T-cell responses

Intracellular cytokine staining (ICS) was used to assess antigen-specific T-cell responses. Cryopreserved PBMC (10^6^ cells) in 200 µl of complete medium were stimulated with either IIV (final 1/100 dilution), nucleoprotein (NP) peptide derived from A/California/04/2009 (H1N1) (122 15–mer peptide overlapping by 11 amino acids) or A/Vietnam/1194/2004 (H5N1) (121 15–mer peptide overlapping by 11 amino acids) at a final concentration of each peptide of 1 µg/ml. NP is the main viral protein recognized by cross-reactive T cells [12]. All stimulated PBMC cultures contained 1 µg/ml of anti-CD28 and 1 µg/ml of anti-CD49. Staphylococcal enterotoxin (SEB) (4 µg/ml) and medium were used as positive and negative controls, respectively. After 2 h of stimulation, Golgiplug was added to inhibit cytokine secretion and the cell cultures were further incubated overnight. Then cells were washed and stained for CD4 and CD8. The stained cells were fixed/permeabilized and intracellular cytokines were stained with MAbs against IFN-g and IL-2. Finally, stained cells were analysed by four-color flow cytometry. The samples considered positive were those in which the percentage of cytokine-staining cells was at least twice that for the background or in which there was a distinct population of bright cytokine-positive cells.

#### Analysis of nasal wash samples

Samples were stored in liquid nitrogen until use. IgA was purified from nasal fluid samples using a *Staphylococcus aureus* superantigen-like protein 7/Agarose column. Nasal wash purified IgA samples were assessed for influenza reactivity in HI and neutralization assays.

### Statistical analysis

This was a descriptive pilot feasibility study, designed to detect trends in safety and immune responses that could warrant expanded investigations of potentially promising combinations [13] [14]. A target sample size of 5 in arms 1 and 2 (homologous prime boost), and 8 subjects in arms 3 and 4 (heterologous prime boost), was selected based on what is generally considered adequate for phase I trials [15]–[17]. Since recruitment of subjects was dependant on a limited number of consenting US citizens resident in Bangkok who fulfilled eligibility criteria, there was no stratification of enrolment based on age or gender; these and other possible confounders can be addressed in any future large-scale trials.

Immunological titres and other continuous data were expressed as geometric means (GMT) and 95% confidence intervals (95%CI), or as medians (interquartile range); differences between vaccine groups were compared using Kruskall-Wallis and Mann-Whitney rank sum non-parametric tests. *In vivo* (AE) data was presented using counts and percentages; differences between doses and vaccine groups were compared using Chi-squared and Fishers exact tests.

All statistical tests were performed at the 5% significance level and corresponding 95% confidence intervals were estimated. Statistical analyses were performed using SPSS version 12.0 (SPSS Inc., Chicago, IL) and Stata version 11 (College Station, TX) and reported using CONSORT methodology [18].

## Results

**Figure 1:**
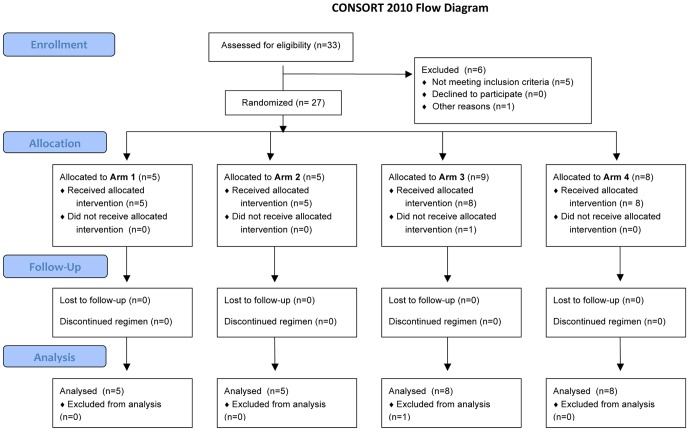
CONSORT (2010) flow diagram.

Between October 2009 and March 2010 twenty-six subjects were enrolled into the study: 5 in each of Arms 1 and 2, and 8 in each of Arms 3 and 4 (Figure 1, Table 1). None gave a prior history of recent (within 6 months) influenza-like illness (ILI) despite emergence of pandemic H1N1 2009 influenza in Bangkok during the study period. This was a highly vaccinated group of subjects, with 73% having received at least one previous influenza vaccination; the median number of influenza vaccinations was 10 (IQR 10, range 0–17) of whom 21% reported experiencing 1 or more side effects after previous vaccination.

**Table 1:** Baseline characteristics in 26 healthy subjects, n (%).

	Group 1 LAIV-LAIV	Group 2 IIV-IIV	Group 3 LAIV-IIV	Group 4 IIV-LAIV	Overall n (% of total)
*Number of subjects*	5	5	8	8	26
*Male:female*	4∶1	1∶4	2∶6	2∶6	9∶17 (35∶65)
*Caucasian*	4	5	7	8	24 (92)
*Age, years, median (IQR)*	38 (1.4)	48 (4.5)	43 (7.7)	33 (6.7)	40 (13.8)
Age range, years					
≤30	1	0	1	4	6 (23)
31–40	3	0	2	3	8 (31)
41–49	1	5	5	1	12 (46)
*No. of previous seasonal influenza vaccinations, median (IQR)*	8 (9)	14 (10)	9 (9)	10 (14)	10 (10)
No.**of previous seasonal influenza vaccinations					
0	0	1	1	5	7 (27)
1–5	2	1	1	1	5 (19)
6–10	1	0	3	1	5 (19)
>10	2	3	2	1	8 (31)
Yes, but number unknown	0	0	1	0	1 (4)
*Received LAIV previously*	1	2	0	1	4/19 (21)
Years since last influenza vaccination					
≤ 2	0	0	0	0	0
≥ 3	5	4	6	3	18 (69)
Unknown	0	0	1	0	1 (4)
Cross-reactive antibodies present at baseline*					
Against H5N1	3	2	5	5	15 (58)
Against pandemic H1N1 2009	0	1	1	7	9 (35)
Against both H5A1 and pandemic H1N1 2009	1	2	3	5	11 (42)
None against H5N1 or pandemic H1N1 2009	1	2	3	1	7 (27)

LAIV = live attenuated influenza vaccine; IIV = inactivated influenza vaccination; * as assessed by haemagglutination inhibition, neutralization and neuraminidase inhibition assays.

### Immunological findings

#### Vaccine–induced antibody responses

Post-vaccination serum antibody titres against seasonal vaccine strains (A/H1N1, A/H3N2 and influenza B) were generally robust following vaccination with IIV as determined by both haemagglutination inhibition (HI) and neuraminidase inhibition (NI) (Figures S1, S2, S3). Increased titres were primarily generated by one dose of IIV, whether given as prime in homologous prime-boost vaccination (Group 2), or as prime or boost in heterologous prime-boost vaccination (Groups 3 and 4). GMT increases of HI antibody for H1N1, H3N2 and influenza B were, respectively: Group 2 ((from 26 to 70, 13 to 121 and 106 to 160; or 2.6-, 9.2- and 1.5-fold increases), Group 3 (from 17 to 80, 14 to 123 and 104 to 247; or 4.8-, 8.0- and 2.4-fold increases), and Group 4 (from 20 to 67, 14 to 160 and 123 to 207; or 3.3-, 11.3- and 1.7-fold increases). IIV did not prime for IIV (Group 2), as GMT values plateaued after the first dose. LAIV (Group 1, 3 and 4), regardless of vaccination schedule, was not associated with any demonstrable serum antibody production. GMT values of IIV prime (Groups 2 and 4), compared with IIV boost (Group 3), indicated LAIV did not prime for IIV, or that it led to serum antibody levels generated by IIV prime (Group 4) being maintained for longer. Influenza B antibodies were relatively high at baseline, and associated with relatively smaller post-vaccination antibody responses after 14 days. Such an inverse correlation was also detected in some subjects who did have high baseline serum HI titres(≥40) to H1N1 and H3N2. Relatively high pre-existing NI titres were detected for A/H1N1 (GMT 280; 95%CI 205 to 382), but not A/H3N2 (GMT 80; 95%CI 80 to 80). IIV as prime or boost (Group 2, 3 and 4) increased GMT for A/H1N1 from 197 to 592, 239 to 1004 and 306 to 1025 (3–4.5 fold increase). For A/H3N2, IIV, as prime or boost increased GMT from 80 to 265, 80 to 145 and 80 to 227 in groups 2, 3 and 4 respectively (1.8–3.3 fold increase).

#### Cross-reactive antibody responses

**Figure 2:**
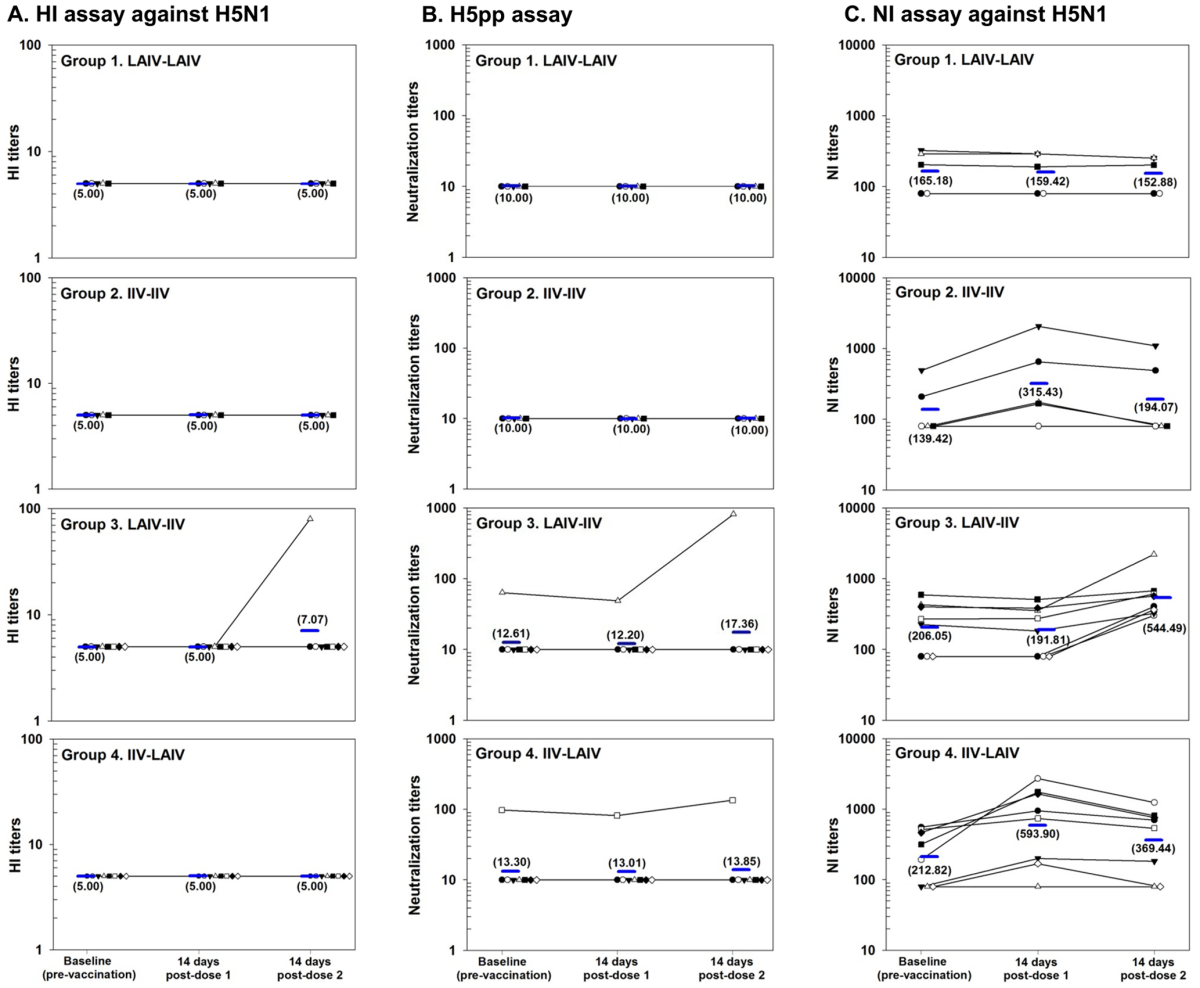
Individual and geometric mean serum haemagglutination inhibition assay, H5pp assay and serum neuraminidase inhibition assay results against avian H5N1 virus in 26 healthy human volunteers measured at baseline and two weeks following each dose of prime boost seasonal influenza vaccination (2 doses administered 8 weeks apart).

**Figure 3:**
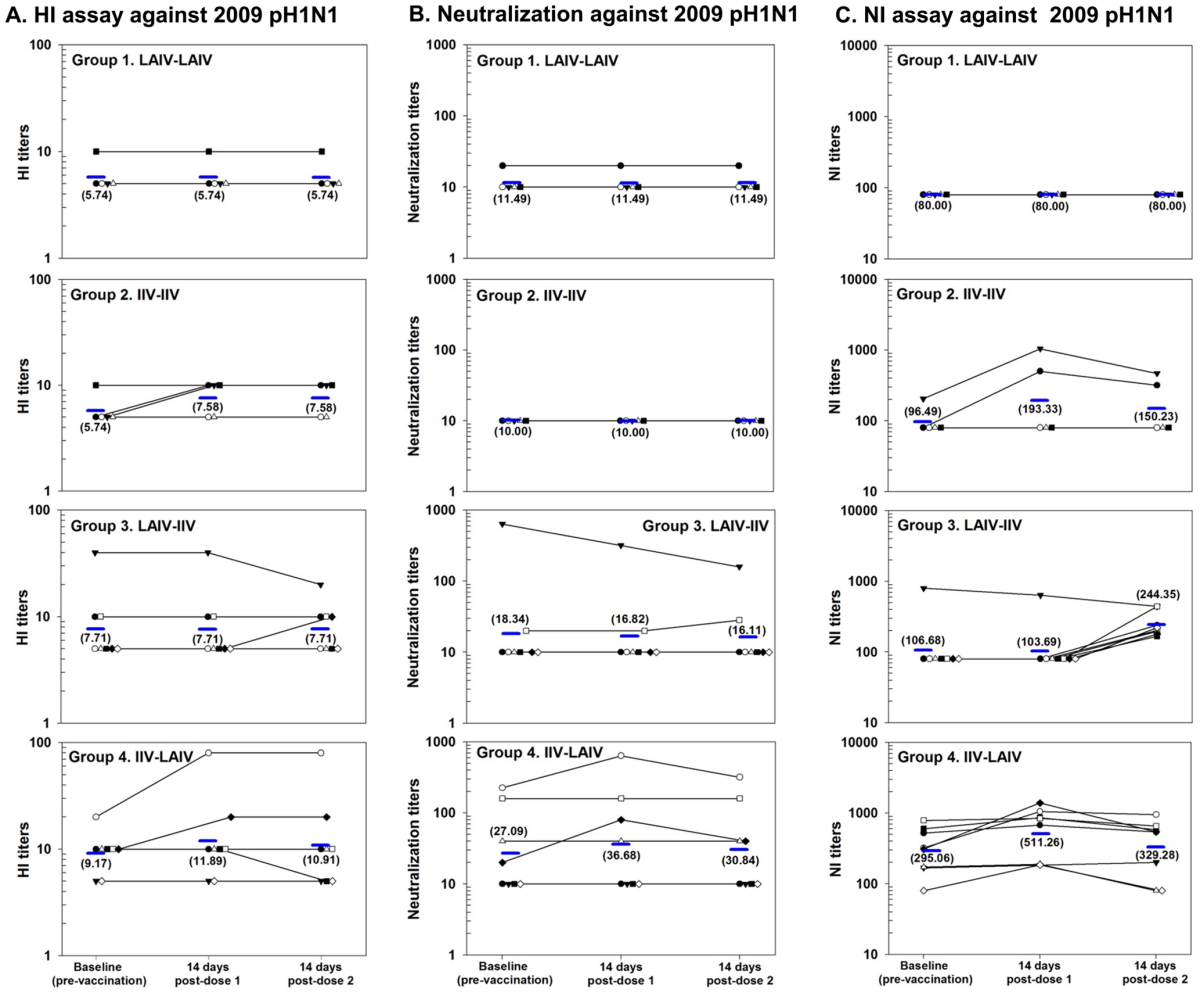
Individual and geometric mean serum haemagglutination inhibition assay, microneutralization assay and serum neuraminidase inhibition assay results against pandemic H1N1 2009 virus in 26 healthy human volunteers measured at baseline and two weeks following each dose of prime boost seasonal influenza vaccination (2 doses administered 8 weeks apart).

These were assessed using HI, NT and NI assays. The induction of cross-reactive antibodies to H5N1 or 2009 pH1N1 was independent of antibody response to vaccine-specific strains (Figures 2 and 3; figures S1, S2, S3). IIV did not prime for IIV (Group 2), and LAIV (Group 1, 3 and 4), regardless of schedule, did not generate serum antibody production, nor prime for IIV (Group 3).

##### H5N1

At baseline 2 of 26 (8%) subjects had cross-reactive neutralizing antibodies against H5N1 by NT (H5pp) assay, but none by HI assay (Figure 2A and B). In general HI and NT antibody titres against H5N1 did not increase significantly after vaccination. However one subject in Group 3 developed marked increased HI (from <10 at baseline to 80) and NT (from 64 to 824) after IIV boost of LAIV prime. A second subject in Group 4, who had a baseline cross-reactive NT titre of 98, did not develop increased NT or HI titres after IIV prime.

At baseline, serum cross-reacting antibodies by NI assay against H5N1 were found in 15 (58%) subjects (Figure 1C). IIV, as prime or boost significantly increased GMT to H5N1 compared to baseline amongst subjects in heterologous prime boost Group 3 (from 206 to 544, 2.6-fold increase, p = 0.04) and non-significantly in heterologous prime boost Group 4 (from 213 to 594, 2.8-fold increase, p = 0.09) and in Group 2 following the first dose of vaccine (from 139 to 315, 2.3-fold increase, p = 0.3). Similar increases were not observed following LAIV.

All serum samples showed reactivity to H5N1 M2e (A/Vietnam/1203/04) expressed on HEK 293 cell line at baseline. However, following vaccination increased M2e binding was negligible regardless of vaccination regimen (data not shown). Purified nasal IgA from all subjects collected both before and after vaccination showed no cross-neutralizing activity against H5N1 (data not shown).

##### 2009 pH1N1

At baseline 42% (11 of 26) subjects had pre-existing cross-reactive antibodies to 2009 pH1N1 detected by HI (range 10–40), and 27% (7 of 26) by NT (range 20–640) assays; of these subjects, 8/11 (73%) and 6/7 (86%) gave a history of previous seasonal influenza vaccination. Post-vaccination HI and NT titre increases against 2009 pH1N1 were generally modest, although an increase in NT titre was more common in subjects with pre-existing cross-reactive antibodies (Figure 3A and B). Single doses of IIV, given as prime (Groups 2 and 4), or as boost (Group 3), were associated with an increase in HI titres for 5 subjects (Group 2∶ 2, Group 3∶ 1; Group 4∶ 2, respectively) and NT titres for 3 subjects (Group 3∶ 1; Group 4∶ 2, respectively). Two of 8 subjects in Group 4 with pre-existing cross-reactive antibodies developed both increased HI (10 to 20, and 20 to 80, respectively) and NT (20 to 80, and 226 to 640, respectively) titres against 2009 pH1N1, after IIV prime.

Using NI, serum cross-reacting antibodies against 2009 pH1N1 were found in 35% subjects at baseline (Figure 3C). IIV, given as prime or boost, significantly increased GMT to 2009 pH1N1 among subjects in Group 3 (from 107 to 244, 2.3-fold increase, p = 0.007) and non-significantly in Groups 2 (from 96 to 193, 2.0-fold increase, p = 0.4) and 4 (from 295 to 511, 1.7-fold increase, p = 0.1). Purified nasal IgA from all subjects collected both before and after vaccination showed no cross-neutralizing activity against 2009 pH1N1 (data not shown).

#### T-cell responses

**Figure 4:**
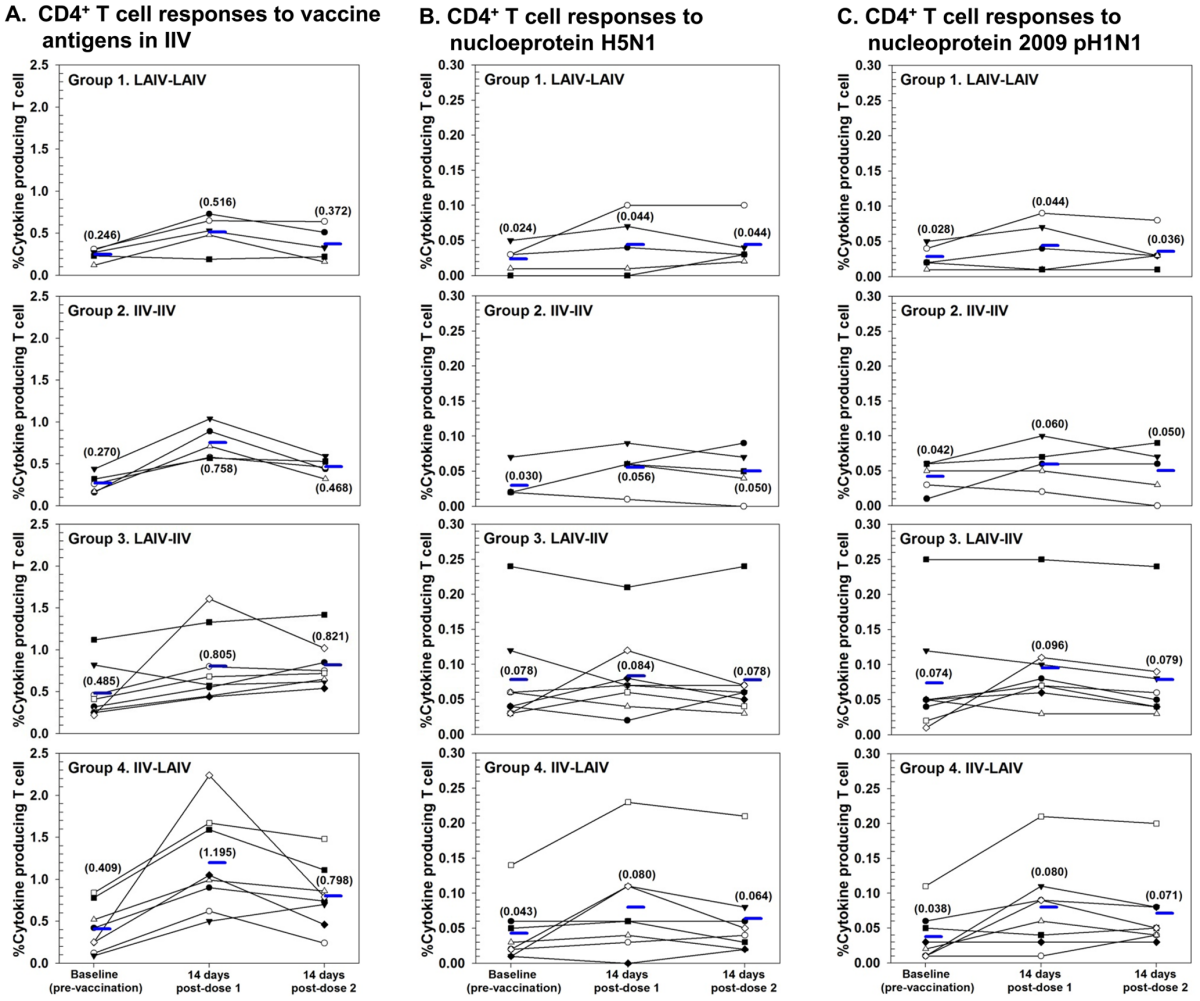
Individual and geometric mean peripheral blood CD4+ T-cell responses measured at baseline, two weeks after dose 1 and two weeks after dose 2, by study group: (A) against vaccine antigens in IIV, (B) against nucleoprotein H5N1, and (C) against nucleoprotein pH1N1 2009.

The magnitude of the T-cell response, defined as the combined frequency of IL-2, IFN-g and IL-2 plus IFN-g producing T-cells is shown in Figure 4. Regardless of vaccination schedule, priming with LAIV or IIV induced specific T-cell responses against IIV antigens in about 80% subjects as demonstrated by an increase of cytokine producing CD4^+^ T-cells, ranging from 2–9 fold. Boosting with either LAIV or IIV failed to further increase the CD4^+^ T-cell response.

Cross-reactive T-cell responses were assessed after *in vitro* stimulation with peptide pools generated from nucleoprotein (NP) of 2009 pH1N1 or H5N1 (A/Viet Nam/1194/2004) (Figure 4B and C). Approximately 40% subjects demonstrated increased cross-reactive CD4^+^ T-cell responses to NP of 2009 pH1N1 and to H5N1. However, these responses were less robust than those directed against IIV antigens. A negligible recall CD8^+^ T-cell response was observed after *in vitro* stimulation with IIV antigens or NP peptide pool (data not shown).

### Safety and tolerability

All vaccine doses were well tolerated, there were no serious adverse events, and no subject had to discontinue study participation early. Thirteen subjects received LAIV and 13 received IIV as Dose 1: six and 10 respectively reported 52 AEs that were considered probably or definitely related to vaccine (Table 2). Overall 168 AEs were reported during the 4-week period following Dose 1, and 171 during the same period after Dose 2.

**Table 2:** Adverse events reported during 28 days following Doses 1 and 2 of trivalent seasonal influenza vaccine: number of adverse events (number of vaccine-attributable adverse events).

	Dose 1	Dose 2
Adverse events	Arms 1 & 4 LAIV	Arms 2 & 3 IIV	Total	Arm 1 LAIV-LAIV	Arm 2 IIV-IIV	Arm 3 LAIV-IIV	Arm 4 IIV-LAIV	Total
Number of subjects receiving vaccine	13	13	26	5	5	8	8	26
Number of subjects reporting ≥1 AE: Total (vaccine-attributable number)	12 (6)	12 (10)	24 (16)	5 (1)	5 (5)	8 (4)	8 (0)	26 (10)
Number of AEs: Total number (vaccine-attributable number)	74 (31)	94 (21)	168 (52)	33 (2)	53 (8)	77 (8)	98 (5)	171 (23)
Injection site								
Bruise	-	1 (0)	1 (0)	-	0	0	-	0
Impaired movement	-	3 (3)	3 (3)	-	2 (2)	0	-	2 (2)
Pain	-	8 (8)	8 (8)	-	4 (4)	4 (4)	-	8 (8)
Swelling	-	0	0	-	1 (1)	1 (1)	-	2 (2)
Systemic								
Fever	4 (2)	3 (2)	7 (4)	1 (0)	0	0	0	1 (0)
Chills	3 (2)	2 (1)	5 (3)	1 (0)	0	0	0	1 (0)
Fatigue	5 (3)	8 (1)	13 (4)	2 (0)	0	2 (1)	7 (1)	11 (2)
Loss of appetite	3 (2)	2 (0)	5 (2)	1 (0)	0	1 (0)	0	2 (0)
Muscle aches	5 (2)	2 (2)	7 (4)	0	2 (0)	0	1 (0)	3 (0)
Respiratory								
Sore throat	7 (5)	3 (0)	10 (5)	2 (0)	1 (0)	6 (0)	7 (2)	16 (2)
Runny nose	7 (3)	13 (0)	20 (3)	6 (0)	6 (0)	5 (0)	12 (1)	29 (1)
Blocked nose	6 (3)	5 (0)	11 (3)	5 (0)	1 (0)	5 (0)	8 (1)	19 (1)
Cough	1 (1)	3 (0)	4 (1)	2 (0)	0	2 (0)	4 (0)	8 (0)
Gastrointestinal								
Nausea	0	6 (1)	6 (1)	1 (1)	3 (0)	0	1 (0)	5 (1)
Vomiting	1 (0)	0	1 (0)	0	1 (0)	0	0	1 (0)
Abdominal pain	1 (0)	3 (0)	4 (0)	0	0	0	0	0
Diarrhoea	4 (0)	2 (0)	6 (0)	0	1 (0)	0	1 (0)	2 (0)
Neurological								
Headache	6 (3)	8 (2)	14 (5)	(1)	0	(1)	0	(2)
Dizziness	1 (1)	1 (0)	2 (1)	0	0	0	0	0

LAIV = live attenuated influenza vaccine; IIV = inactivated influenza vaccination; AE = adverse event.

After Dose 2 there was no obvious increase in frequency of occurrence of AEs. Two of the 5 subjects who received 2 doses of IIV (Group 2) complained of more severe pain at the injection site following Dose 2 than following Dose 1 even though Dose 2 was administered into the other arm. The two heterologous prime boost regimens were not associated with any excess of reported AEs compared to the homologous prime boost regimens although the numbers in all groups are very small. There were no serious adverse events (SAEs) reported in this trial.

## Discussion

This is the first prospective study in humans to test the hypothesis that prime boost regimens of seasonal influenza vaccine can be used to elicit production of sub-heterotypic antibodies. Our findings show that both homologous and heterologous prime boost immunization did not enhance serum HI and NT titres to 2009 pH1N1 and avian H5N1 as compared to a single dose of vaccine. Interestingly the increase of NT titres to both viruses was observed in subjects who had pre-existing cross-reactive antibodies at baseline; this supports previously reported findings [19]. The observations provide more evidence that generation of cross-reactive antibodies are derived from activation of cross-reactive memory B-cells that recognize conserved epitopes in multiple influenza strains [20] [21]. However, this type of immunological response seems to be sporadic and may be influenced by genetic and/or viral factors. Several additional subjects, including one with a high baseline H5pp response, failed to enhance antibody titres further following seasonal vaccination. More work is needed to understand the complex factors involved in generation of cross-reactive antibody responses in given individuals.

Unlike IIV, LAIV induced modest serum antibody responses in all subjects regardless of whether LAIV was used as a prime or as a boost. These observations are in line with previous reports, which demonstrated modest serum HI titres after LAIV nasal vaccination [3] [22]. The biological mechanisms for protective immunity elicited by LAIV are believed to involve a local immune response in the lung via mucosal antibodies and T-cell responses [3]. Recent reports have suggested a role for memory B cells localised to the lung conferring protection against disease [23].

Neuraminidase activity is required for the release of newly budded virus from the infected cell surface. Studies in humans suggest that antibody to influenza neuraminidase is associated with resistance to clinical disease [24] [25]. In this study, serum NI titres against H5N1 and 2009 pH1N1 increased significantly in heterologous prime boost Group 3 (LAIV-IIV). However, it would be premature to draw conclusions from this since the sample size in this study was very small and more subjects in this group had detectable antibodies at baseline than in other groups.

Our findings confirm the efficacy of single dose trivalent seasonal influenza vaccine in conferring robust antibody responses against vaccine viruses in healthy adults. The addition of a second (booster) dose in either homologous regimen (Groups 1 and 2) conferred no demonstrable benefit. Reassuringly the addition of the second dose had no impact on adverse event reporting in this group of healthy adults. The only observation of note was that 2 of 5 subjects in Group 2 complained of more intense pain following the second dose of IIV. A similar finding was observed in a study of healthy children aged 5–8 years [26] where a significantly higher proportion of subjects reported pain following the second dose. Both prime boost regimens were also well tolerated with no excess of AEs after the second dose, including IIV given as Dose 2.

Both IIV and LAIV elicited recall CD4^+^ T-cell responses against vaccine antigens as detected by ICS staining of IFN-g and IL-2. In agreement with previous findings [19] [27] [28] [29] we detected pre-existing cross-reactive CD4^+^ T-cells specific to NP derived from 2009 pH1N1 and H5N1. The increases in cross-reactive T-cell responses were observed in some subjects but the prime boost vaccination regimen did not appear to result in superior cross-reactive T- cell responses. Unlike in the mouse model [30] [31] [32] the role of cross-reactive T-cells in protecting against influenza has not been well described in humans. A recent human challenge study with influenza demonstrated that pre-existing CD4^+^ T-cell responses to conserved NP and matrix protein could reduce severe illness in the absence of specific antibodies [33]. One could speculate that cross-reactive T-cells may mediate heterosubtypic immunity in humans as well. Further studies are required to support this hypothesis.

This study was a pilot feasibility study and as such had a number of important limitations. Firstly the sample sizes are small and therefore unlikely to detect subtle trends or immunological responses generated in just a small proportion of subjects. The small size was off-set by having a relatively highly vaccinated group of subjects, with a high proportion of females, both of which were considered to increase the chances that favourable immunological responses would be observed [34] [35]. Secondly the study took place during the initial stages of the 2009 H1N1 influenza pandemic, which had just reached Bangkok at the time the study commenced. Although none of the subjects developing cross-protective responses to pH1N1 2009 during the course of the study gave a history of influenza-like illness, it remains a possibility that the observed responses against pH1N1 2009 were in fact influenced by *in vivo* exposure to natural disease. Even if this is the case, it cannot explain the robust serum antibody response to H5N1 observed in the single subject from Group 3. Finally the lack of good correlates of protection generated by LAIV discussed above may mean that we missed a cross-protective effect afforded by this vaccine given either as prime or boost.

Despite the fact that broad immune responses indicating significant cross-protection against pH1N1 2009 and avian H5N1 influenza viruses were not observed in the majority of prime boost recipients, *in vitro* cross-protection against one or other virus assessed by HI and/or NT was observed in several individuals; moreover most subjects developed detectable responses on NI assay and some developed cross-reactive CD4^+^ T cell responses against nucleoprotein, These results lend support to the current recommendation for administration of seasonal influenza vaccine at the outset of an influenza pandemic. Such a strategy may afford some protection to a sub-set of individuals, particularly those with a degree of pre-existing immunity. Our data support the administration a single dose of trivalent seasonal vaccine to those individuals at risk of disease who have not previously received that season's recommended seasonal influenza vaccine; however, based on our preliminary findings we do not have enough evidence to recommend administration of a second (prime boost) dose. Even if progression to clinical or severe disease is prevented in only a minority of recipients, seasonal influenza vaccine administration at the outset of a pandemic could represent a cost-effective public health strategy and buy valuable time while a specific vaccine is being developed.

## Supporting Information

Figure S1 Individual and geometric mean serum assay results against seasonal influenza A/H1N1 vaccine virus in 26 healthy human volunteers measured at baseline and two weeks following each dose of prime boost seasonal influenza vaccination (2 doses administered 8 weeks apart). (A) haemagglutination inhibition, and (B) neuraminidase inhibition assay.
(TIFF)

Figure S2 Individual and geometric mean serum assay results against seasonal influenza A/H3N2 vaccine virus in 26 healthy human volunteers measured at baseline and two weeks following each dose of prime boost seasonal influenza vaccination (2 doses administered 8 weeks apart). (A) haemagglutination inhibition, and (B) neuraminidase inhibition assay.
(TIFF)

Figure S3 Individual and geometric mean serum haemagglutination inhibition assay results against seasonal influenza B vaccine virus in 26 healthy human volunteers measured at baseline and two weeks following each dose of prime boost seasonal influenza vaccination (2 doses administered 8 weeks apart).
(TIFF)

Protocol S1 Study Protocol.
(PDF)

Checklist S1 CONSORT (2010) checklist.
(DOC)
