# Supplementary figures and images for: Evaluation of In Vitro Cross-Reactivity to Avian H5N1 and Pandemic H1N1 2009 Influenza Following Prime Boost Regimens of Seasonal Influenza Vaccination in Healthy Human Subjects: A Randomised Trial
Source: PLoS One. 2013 Mar 26;8(3):e59674. doi: 10.1371/journal.pone.0059674 (PMC3608534; doi:10.1371/journal.pone.0059674)

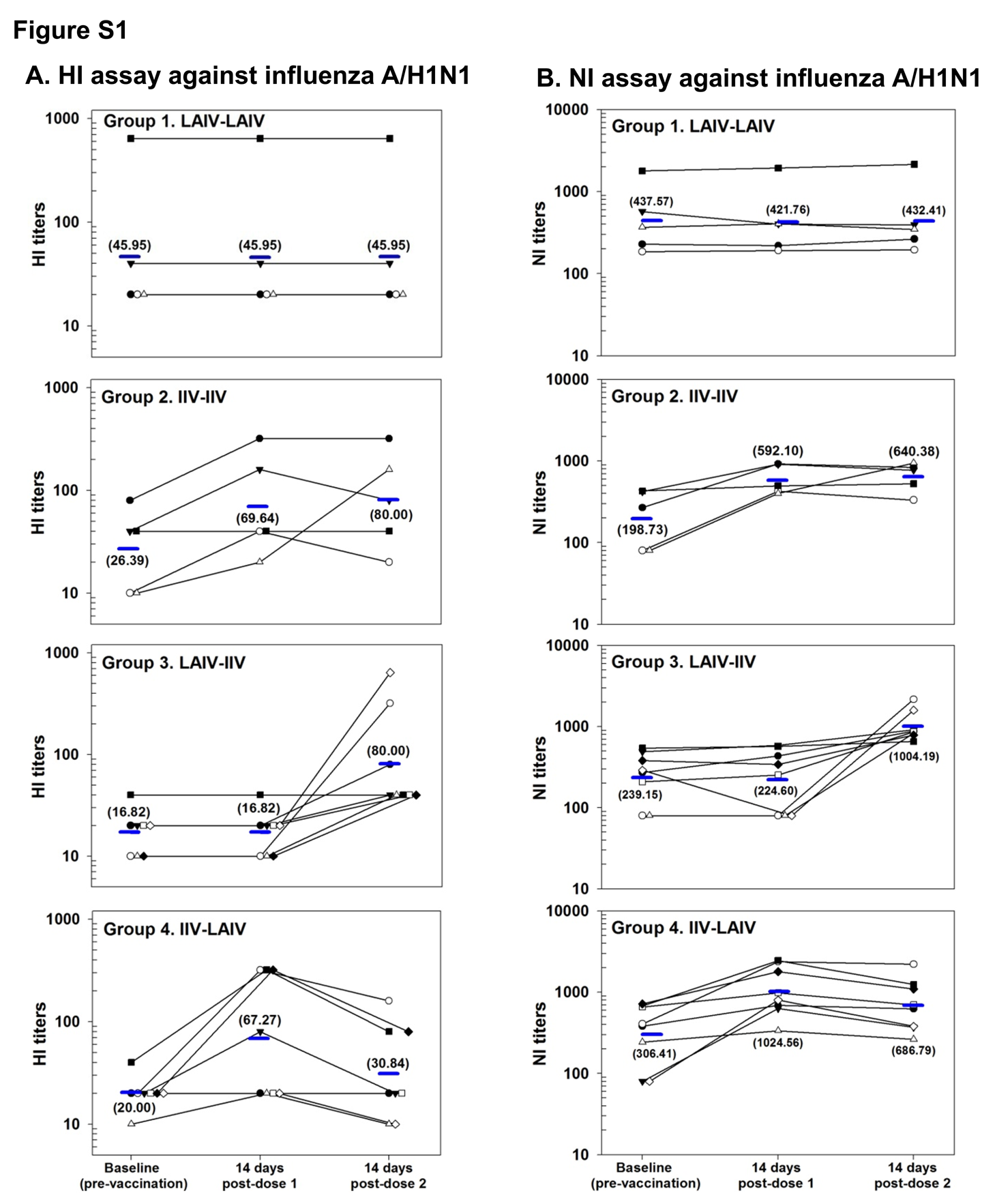

Supplement: Figure S1 — Individual and geometric mean serum assay results against seasonal influenza A/H1N1 vaccine virus in 26 healthy human volunteers measured at baseline and two weeks following each dose of prime boost seasonal influenza vaccination (2 doses administered 8 weeks apart). (A) haemagglutination inhibition, and (B) neuraminidase inhibition assay. (TIFF) [file pone.0059674.s001.tiff]

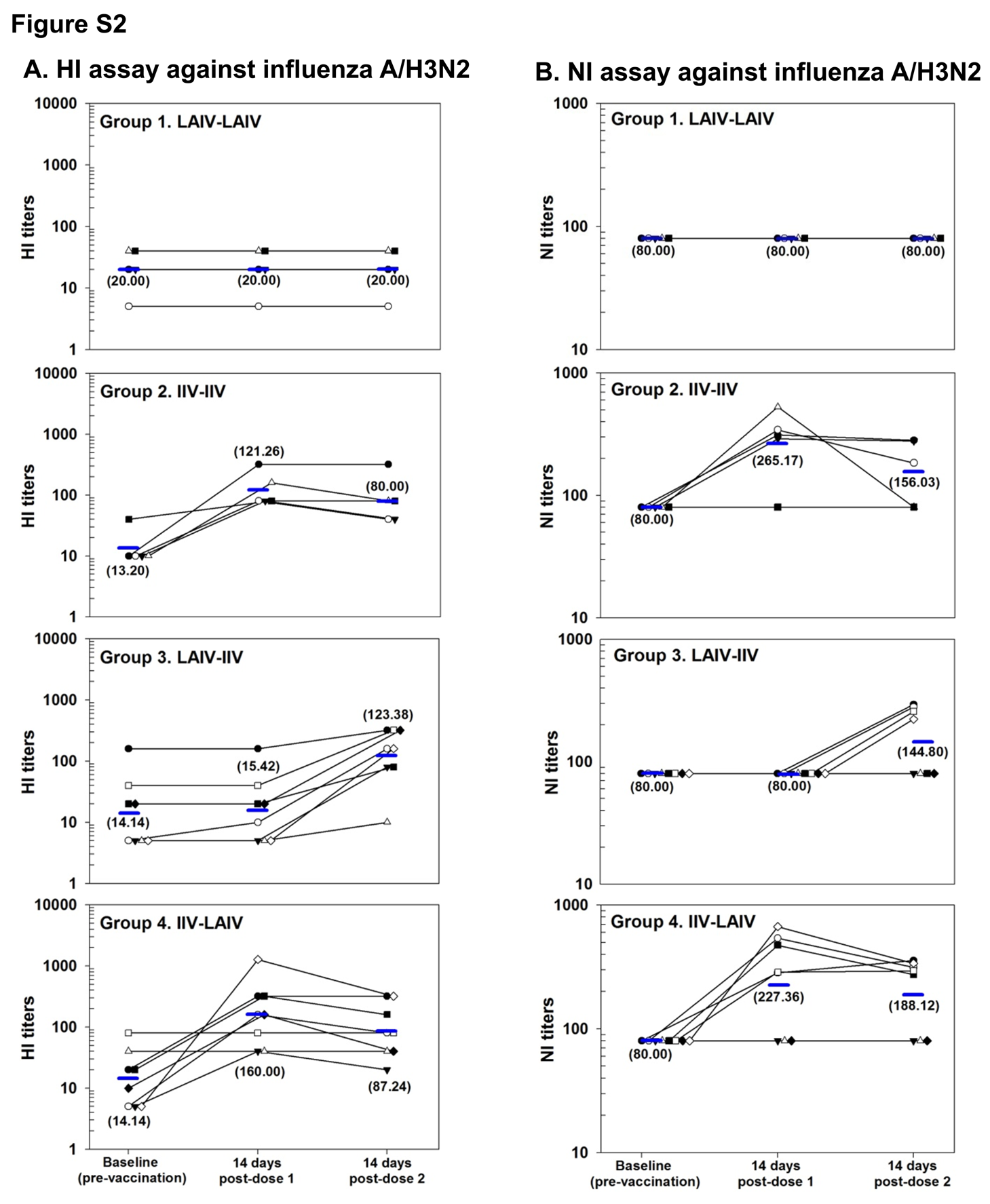

Supplement: Figure S2 — Individual and geometric mean serum assay results against seasonal influenza A/H3N2 vaccine virus in 26 healthy human volunteers measured at baseline and two weeks following each dose of prime boost seasonal influenza vaccination (2 doses administered 8 weeks apart). (A) haemagglutination inhibition, and (B) neuraminidase inhibition assay. (TIFF) [file pone.0059674.s002.tiff]

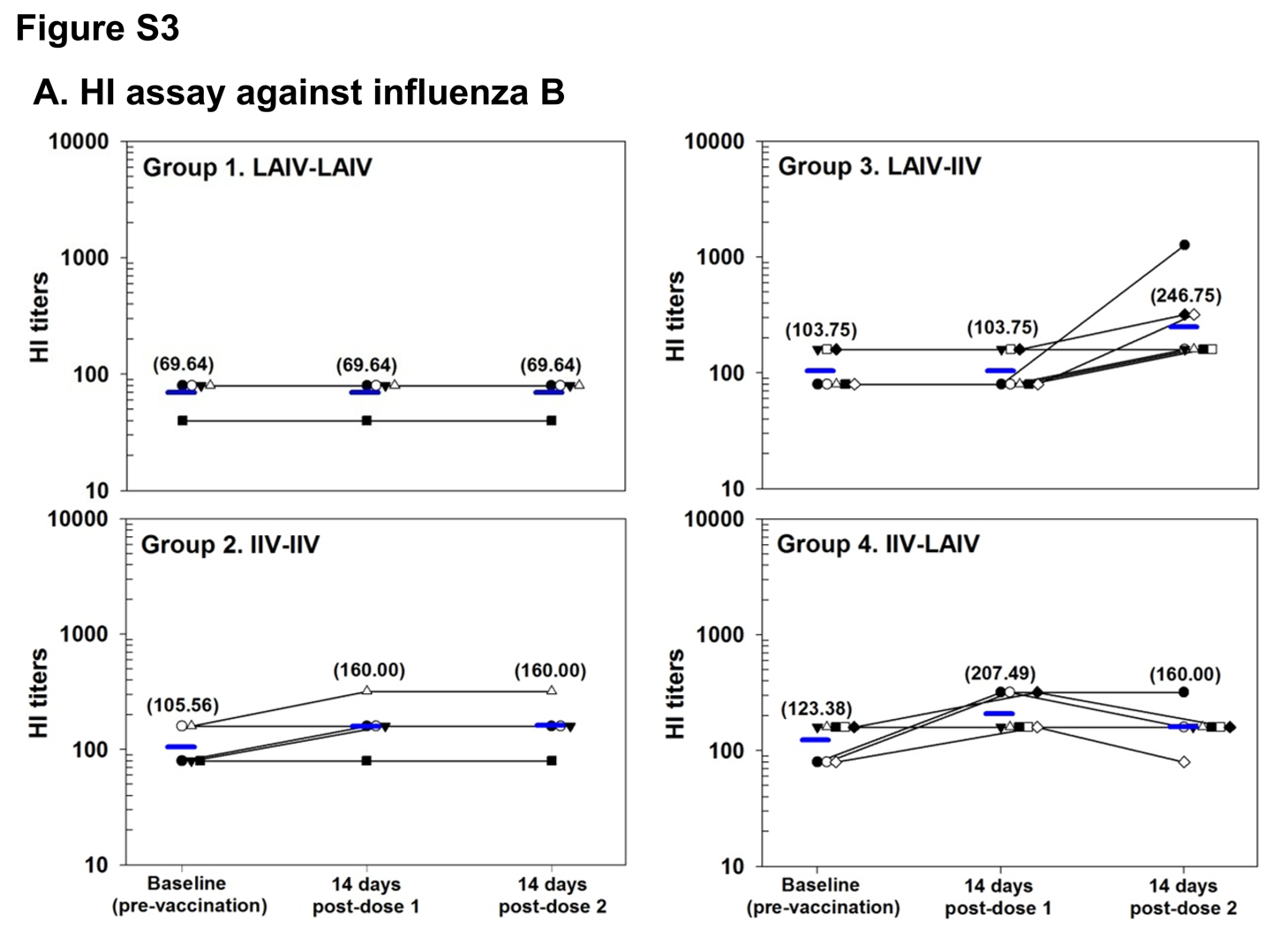

Supplement: Figure S3 — Individual and geometric mean serum haemagglutination inhibition assay results against seasonal influenza B vaccine virus in 26 healthy human volunteers measured at baseline and two weeks following each dose of prime boost seasonal influenza vaccination (2 doses administered 8 weeks apart). (TIFF) [file pone.0059674.s003.tiff]
