# Supplementary material for: Evaluation of In Vitro Cross-Reactivity to Avian H5N1 and Pandemic H1N1 2009 Influenza Following Prime Boost Regimens of Seasonal Influenza Vaccination in Healthy Human Subjects: A Randomised Trial
Source: PLoS One. 2013 Mar 26;8(3):e59674. doi: 10.1371/journal.pone.0059674 (PMC3608534; doi:10.1371/journal.pone.0059674)

Evaluation of *In Vitro* Cross-Reactivity with Avian Influenza H5N1 Virus in Healthy Volunteers Vaccinated with a Prime Boost Regimen of Seasonal Influenza Vaccine

Version Number: 6.0

Short Title: Cross-reactivity of prime boost seasonal influenza vaccine

Protocol Number
WRAIR #1525
HRPO Log #15223

Date: 29 April 2011

1. Study Summary

Objectives

The primary objectives of this study are:

1. To establish whether a prime boost strategy using seasonal LAIV and IIV can augment cross-reactivity with H5N1 and human influenza strains.
2. To detect the presence of antibodies that can inhibit infection with H5N1 and human influenza strains in microneutralization, plaque reduction and H5 pseudotyped lentiviral particle-based assays after vaccination with seasonal LAIV and IIV.
3. To establish the breadth of antibody responses to H5N1 strains and human influenza strains using ELISA, microneutralization, plaque reduction, H5 pseudotyped lentiviral assays, neuraminidase inhibition assay, and hemagglutination inhibition after vaccination with seasonal LAIV and IIV.
4. To observe T-cell responses following vaccination with seasonal LAIV and IIV.
5. To attempt to generate human monoclonal antibodies that are cross-reactive to enzyme neuraminidase and hemagglutinin of H5N1 and pandemic H1N1 2009 in order to investigate further the human response to influenza vaccination

Secondary objectives are to describe the adverse event profile of 2 doses of influenza vaccine given approximately 8 weeks apart in healthy adult volunteers.

Subject Population

Subjects will be healthy adult U.S. citizens residing in Bangkok. FluMist® vaccine is not yet licensed for use in Thailand; hence Thai subjects will not be recruited into this study.

Study Site

The *in vivo* phase of the study will take place at the U.S. Embassy Medical Unit, Bangkok, Thailand. In selected cases individuals may elect to have scheduled follow-up visits (which do not involve vaccination) performed at AFRIMS. Immunology testing will be done at the Department of Immunology and Medicine, AFRIMS and the Faculty of Medicine, Siriraj Hospital, Bangkok.

Number of Subjects

Up to 34 subjects will be recruited: 26 subjects for randomization and a further 8 to act as replacements in case of drop-outs.

Vaccines Administered

Subjects will be randomized to receive one of the following 4 regimens. For each regimen the two doses of vaccine will be separated by 8 weeks (±7 days).

Arm 1: FluMist® live, intranasal vaccine 0.2mL (0.1mL per nostril): 2 doses separated by 8 weeks (±7 days); n=5 subjects. Autologous prime boost regimen 1.

Arm 2: Fluzone® inactivated influenza virus vaccine 0.5mL intramuscularly: 2 doses separated by 8 weeks (±7 days); n=5 subjects. Autologous prime boost regimen 2.

Arm 3: FluMist® live, intranasal vaccine 0.2mL (0.1mL per nostril) single dose, followed by Fluzone® inactivated influenza virus vaccine 0.5mL intramuscularly 8 weeks (±7 days) later; n=8 subjects. Heterologous prime boost regimen 1

Arm 4: Fluzone® inactivated influenza virus vaccine 0.5mL intramuscularly single dose, followed by FluMist® live, intranasal vaccine 0.2mL (0.1mL per nostril) 8 weeks (±7 days) later; n=8 subjects. Heterologous prime boost regimen 2

Study Design and Methodology

This will be a randomized, open-label, pilot feasibility study of four 2-dose vaccine regimens using two commercially available seasonal influenza vaccines to compare immune responses and *in vitro* cross-reactivity against H5N1. Vaccine doses will be spaced by approximately 8 weeks to allow for optimal prime boost conditions. Humoral, cellular and secretory immune responses will be measured 2 and 4 weeks after each vaccine dose and compared with baseline values.

Study Endpoints

The primary study endpoints will be determination of pre and post vaccination immune responses in 26 healthy human subjects to 2 doses of seasonal influenza vaccine including whether there is *in vitro* cross-reactivity against H5N1 virus as follows:

1) Antibodies ELISA titers calculated as the dilution factor required to give an optical density of 0.5.

2) Hemagglutination inhibition activity defined as the titer required to completely inhibit hemagglutination);

3) Microneutralization assay endpoint titer; determined by using 50% specific signal calculation. The endpoint titer will be expressed as the reciprocal of the highest dilution of serum with A490 value less than X, where X = [(average A490 of VC wells) – (average A490 of CC wells)] / 2 + (average A490 of CC wells).

4) Neuraminidase inhibition assay titer defined as the highest sample dilution that produces 50% inhibition of virus control.

5) Plaque reduction assay titer assessed as the highest sample dilution that will give a reduction of plaque count of 50%.

6) H5 pseudotyped lentiviral particle-based serological assay titer assessed as the highest sample dilution that produces 50% inhibition of virus control.

7) Influenza specific T-cell cytokine production by influenza-specific T-cells defined as the percentage of cytokine-staining cells at least twice background.

8) Human monoclonal antibody production from peripheral blood memory B cells isolated from selected subjects

Duration of Subject Participation

This study will take up to 12 months from enrollment of the first subject to completion of the *in vivo* portion. Each subject will be followed up for approximately 12 weeks following entry into the study (screening, dosing, follow-up). The total study duration will be 2 years.

A subset of previously enrolled subjects may be invited to participate in an additional phase of the study, which will involve consent for use of stored sample and a possible single blood draw if extra sample is required. No follow-up will be required after the day of blood draw

Safety Data Collected

Subjects will complete a symptom diary for 14 days following each dose of vaccine. Adverse event monitoring will continue throughout the study duration.

TABLE OF CONTENTS

I. GENERAL INFORMATION 9
II. INVESTIGATOR PROTOCOL AGREEMENT PAGE 11
III. ABBREVIATIONS 12
IV. BACKGROUND INFORMATION 13
IV.1. INTRODUCTION 13
IV.2. IMMUNOLOGY OF INFLUENZA AND INFLUENZA VACCINES 13
IV.2.1. INFLUENZA VIRUS 13
IV.2.2. AVIAN INFLUENZA H5N1 14
IV.2.3. IMMUNE RESPONSES TO INFLUENZA VIRUS 15
IV.2.4. INFLUENZA VACCINES 15
IV.2.5. LIVE ATTENUATED INFLUENZA VACCINES 16
IV.2.6. VACCINES AGAINST AVIAN INFLUENZA H5N1 16
IV.3. FLUMIST® LIVE, ATTENUATED INFLUENZA VACCINE 18
IV.3.1. PRODUCT INFORMATION 18
IV.3.2. SAFETY AND TOLERABILITY FOLLOWING SINGLE AND DUAL DOSE ADMINISTRATION IN HEALTHY SUBJECTS 19
IV.3.3. SAFETY IN SPECIAL GROUPS 20
IV.3.4. SECONDARY TRANSMISSION OF VACCINE VIRUS 21
IV.4. FLUZONE® INFLUENZA VACCINE 22
IV.4.1. PRODUCT INFORMATION 22
IV.4.2. SAFETY AND TOLERABILITY FOLLOWING SINGLE AND DUAL DOSE ADMINISTRATION IN HEALTHY SUBJECTS 22
IV.4.3. SAFETY IN SPECIAL GROUPS 23
IV.5. CONCOMITANT ADMINISTRATION OF FLUMIST® AND FLUZONE® 23
IV.6. STUDY JUSTIFICATION 24
IV.7. ADDITIONAL BACKGROUND INFORMATION AND STUDY JUSTIFICATION FOR AMENDMENT 6 24
V. OBJECTIVES 25
VI. METHODS 25

VI.1. STUDY ENDPOINTS 25

VI.2. STUDY DESIGN 25

VI.3. STUDY SITE 26

VI.4. PATIENT POPULATION 26

VI.5. RECRUITMENT 26

VI.6. RANDOMIZATION 26

VI.7. VACCINATION REGIMENS 26

VI.8. PACKAGING, LABELING AND STORAGE OF VACCINES 27

VI.9. DURATION OF SUBJECT PARTICIPATION 27

VI.10. PROCEDURES 28

VI.10.1. *IN VIVO* PROCEDURES 28

VI.10.1.1. INFORMED CONSENT PROCESS 28

VI.10.1.2. PRE-STUDY ENROLLMENT SCREENING 28

VI.10.1.3. SCHEDULED VISITS 28

VI.10.1.4. UNSCHEDULED VISITS 29

VI.10.1.5. SUMMARY TABLE OF VOLUNTEER ASSESSMENTS 30

VI.10.2. LABORATORY PROCEDURES 30

VI.10.2.1. INFLUENZA VIRUSES 30

VI.10.2.2. DETECTION OF INFLUENZA-SPECIFIC IGA, IGM AND IGG ANTIBODIES BY ELISA 30

VI.10.2.3. HEMAGGLUTINATION INHIBITION ASSAY 31

VI.10.2.4. MICRONEUTRALIZATION ASSAY 31

VI.10.2.5. NEURAMINIDASE INHIBITION ASSAY 31

VI.10.2.6. PLAQUE REDUCTION ASSAY 32

VI.10.2.7. CYTOKINE PRODUCTION BY INFLUENZA-SPECIFIC T-CELLS 32

VI.11. STOPPING RULES 33

VI.12. ACCOUNTABILITY PROCEDURES 33

VI.13. MAINTENANCE OF RANDOMIZATION CODES AND PROCEDURES FOR BREAKING CODES 33

VII. SELECTION AND WITHDRAWAL OF SUBJECTS 33

VII.1. SUBJECT INCLUSION CRITERIA 33

VII.2. SUBJECT EXCLUSION CRITERIA 33

VII.3. SUBJECT WITHDRAWAL CRITERIA………………………………………………. 34

VII.3.1. CONDITIONS FOR SUBJECT WITHDRAWAL…... 34

VII.3.2. DATA TO BE COLLECTED FOR WITHDRAWN SUBJECTS…………………… 34

VII.3.3. FOLLOW-UP OF WITHDRAWN SUBJECTS…………………………………….. 34

VII.3.4. REPLACEMENT OF WITHDRAWN SUBJECTS ………………………………… 34

VIII. CONCOMITANT MEDICATIONS…………………………………………………………….. 35

IX. ASSESSMENT OF IMMUNE RESPONSE…………………………….................................. 35

IX.1. IMMUNE RESPONSE PARAMETERS……………………………..…………………… 35

IX.2. METHODS FOR ASSESSING IMMUNE RESPONSE……………….………………… 35

X. BIOSURETY CONSIDERATIONS …………………………………………………………… 35

XI. SAFETY………………………………………………………………………………………… 35

XI.1. ADVERSE EVENTS…………………………………………………….......................... 35

XI.2. SERIOUS ADVERSE EVENTS……………………………………………………………... 36

XI.3. DETECTION OF AES AND SAES………………………………………………………… 36

XI.4. EVALUATION OF AES………………………………………….………………………… 37

XI.5. FOLLOW-UP OF AES AND SAES…………………………………........................... 37

XI.6. POST-STUDY AES AND SAES…………………………………………………………….. 37

XI.7. REPORTING OF AES AND SAES AND UNANTICIPATED PROBLEMS …………….. 38

XI.8. MONITORING………………………………………………………………………………….. 38

XII. STATISTICAL METHODS……………………………………………………………………… 39

XII.1. SAMPLE SIZE AND POWER ………………………………………………………… 39

XII.2. STUDY OUTCOME ANALYSIS ……………………………………………………… 39

XIII. ETHICAL CONSIDERATIONS…………………………………………………………………… 39

XIII.1. STUDY APPROVAL ……………………………………………………………………… 39

XIII.2. RISKS AND BENEFITS OF PARTICIPATING …………………………………….. 39

XIII.3. BLOOD DRAWS ………………………………………………………………………… 41

XIII.4. SAMPLE STORAGE AND DONATION FOR FUTURE USE……………………… 41

XIV. DATA HANDLING AND RECORD KEEPING……………………………………………… 41

XIV.1. RECORDING OF DATA…………………………………..…………………… 41

XIV.2. CONFIDENTIALITY AND ACCESS TO DATA ………………………………….. 41

XV. QUALITY CONTROL AND QUALITY ASSURANCE………………..…………………… 42

XVI. COMPENSATION……………………………………………………………………….. 42

XVII. FINANCING AND INSURANCE…………………………………………………………. 43

XVIII. ROLES AND RESPONSIBILITIES…………………………………………………………. 43

XIX. CONDITIONS FOR MODIFYING OR TERMINATING THE PROTOCOL ……………… 44

XX. REFERENCES …………………………………………………………………………. 46

APPENDICES ……………………………………………………………………………… 51

APPENDIX 1: FLOW DIAGRAM OF VACCINE COMBINATIONS ……………………. 51

APPENDIX 2: FLOW CHART OF SUBJECT ASSESSMENTS ………..………………… 52

I. GENERAL INFORMATION

Title:	Evaluation of *In Vitro* Cross-Reactivity with Avian Influenza H5N1 Virus in Healthy Volunteers Vaccinated with a Prime Boost Regimen of Seasonal Influenza Vaccine
Study Identifiers:	WRAIR: #1525
Sponsor:	Department of Immunology and Medicine, AFRIMS

Institutions: Armed Forces Research Institute of Medical Sciences (AFRIMS)
Dept. of Immunology and Medicine
315/6 Rajvithi Road, Bangkok 10400, Thailand
Tel. 66-2-696-2700; Fax 66-2-644-4784

Principal Investigator: Delia Bethell, B.M. BCh, MRCPCH.
Dept. of Immunology and Medicine
Armed Forces Research Institute of Medical Sciences (AFRIMS)
315/6 Rajvithi Road, Bangkok 10400, Thailand
Tel. 66-2-644-5775, Fax 66-2-644-4784
Email: delia.bethell@afrims.org

Associate Investigators:
In vivo procedures: David Saunders, M.D., LTC, MC
Dept. of Immunology and Medicine
Armed Forces Research Institute of Medical Sciences (AFRIMS)
315/6 Rajvithi Road, Bangkok 10400, Thailand
Tel. 66-2-644-5775, Fax 66-2-644-4784
Email: david.saunders@afrims.org

Stephen Thomas, M.D., FACP, LTC.
Dept. of Virology
Armed Forces Research Institute of Medical Sciences (AFRIMS)
315/6 Rajvithi Road, Bangkok 10400, Thailand
Tel. 66-2-644-4888, Fax 66-2-644-4760
Email: stephen.thomas@afrims.org

Douglas Walsh, M.D., COL, MC
Dept. of Immunology and Medicine
Armed Forces Research Institute of Medical Sciences (AFRIMS)
315/6 Rajvithi Road, Bangkok 10400, Thailand
Tel. 66-2-644-5775, Fax 66-2-644-4784
Email: douglas.walsh@afrims.org

In vitro procedures: Sathit Pichyangkul, Ph.D.
Dept. of Immunology and Medicine

Armed Forces Research Institute of Medical Sciences (AFRIMS)
315/6 Rajvithi Road, Bangkok 10400, Thailand
Tel. 66-2-644-5775, Fax 66-2-644-4784
Email: sathitP@afrims.org

Stuart Tyner, Ph.D.
Dept. of Immunology and Medicine
Armed Forces Research Institute of Medical Sciences (AFRIMS)
315/6 Rajvithi Road, Bangkok 10400, Thailand
Tel. 66-2-644-5775, Fax 66-2-644-4784
Email: stuart.tyner@afrims.org

Protocol Consultant: Mark Fukuda, M.D., LTC(P), MC
Armed Forces Health Surveillance Center
2900 Linden Lane, Suite 200
Silver Spring, MD 20910
Tel. 301-319-3289
Fax. 301-319-9213
Email: mark.m.fukuda@us.army.mil

Medical Monitor: Robert V. Gibbons, M.D. LTC
Dept. of Virology
Armed Forces Research Institute of Medical Sciences (AFRIMS)
315/6 Rajvithi Road, Bangkok 10400, Thailand
Tel. 66-2-644-4888, Fax 66-2-644-4760
Email: robert.gibbons@afrims.org

Contracted Laboratories: 1. BIOTECH
National Center for Genetic Engineering and Biotechnology
113, Thailand Science Park
Pathumthani 12120, Thailand

2. Mahidol University:

a. Department of Microbiology
Faculty of Medicine
Siriraj Hospital
Bangkok 10700, Thailand
Tel. 66-2-419-7059, Fax. 66-2-418-4148

b. Department of Microbiology
Faculty of Science
Bangkok 10400, Thailand
Tel. 66-2-201-5528, Fax. 66-2-644-5411

II. Investigator Protocol Agreement Page

1. I agree to follow this protocol version as approved by the IRBs/ERCs.

2. I will conduct the study in accordance with applicable IRB/ERC requirements, federal regulations, and state and local laws to maintain the protection of the rights and welfare of study participants.

3. I certify that I, and the study staff, have received the requisite training to conduct this research protocol.

4. I will not modify the protocol without first obtaining an IRB/ERC approved amendment and new protocol version unless it is necessary to protect the health and welfare of study participants.

5. In accordance with Command Policy 2008-35, I will ensure that the Commanding General receives a pre-brief (Executive) Summary and approves the study prior to execution.

6. I will ensure that the data (and/or specimens) are maintained in accordance with the data (and/or specimen) disposition outlined in the protocol. Any modifications to this plan should first be reviewed and approved by the applicable IRBs/ERCs.

7. I will promptly report changes to the research or unanticipated problems to the WRAIR IRB immediately via the WRAIR Division of Human Subjects Protection at (301) 319-9940 (during duty hours) or to the WRAIRDHSP@amedd.army.mil and submit a written report within 10 working days of knowledge of the event.

8. I will prepare continuing review reports at an interval established by the IRB/ERC, and a study closure report when all research activities are completed.

9. I will immediately report to the WRAIR Division of Human Subjects Protection knowledge of any pending compliance inspection by any outside governmental agency.

10. I agree to maintain adequate and accurate records in accordance with IRB policies, federal, state and local laws and regulations.

Signed: ______________________________ Date: __________________________

Name: Dr. Delia Bethell

III. ABBREVIATIONS

AE	Adverse event
AFRIMS	Armed Forces Research Institute of Medical Sciences
B-HCG	Beta human chorionic gonadotropin
BP	Blood pressure
CBC	Complete blood count
CTL	Cytotoxic T-lymphocyte
CRC	Clinical Research Coordinator
CRF	Case report form
DBRPC	Double-blind randomized placebo-controlled trial
DHSP	Division of Human Subjects Protection
ELISA	Enzyme-linked immunosorbant assay
FDA	Food and Drug Administration
FWA	Federal-Wide Assurance
GCP	Good Clinical Practices
HA	Hemagglutinin
HIV	Human immunodeficiency virus
HR	Heart rate
HSRRB	Human Subjects Research Review Board
ICH	International Conference on Harmonization
IgA	Immunoglobulin A
IgG	Immunoglobulin G
IgM	Immunoglobulin M
IRB	Institutional Review Board
IIV	Inactivated influenza vaccine
LAIV	Live attenuated influenza vaccine (FluMist®)
NA	Neuraminidase
NIH	National Institute of Health
PBMC	Peripheral blood mononuclear cells
PE	Physical examination
PI	Principal Investigator
QC/QA	Quality control/Quality assurance
RR	Respiratory rate
SAE	Serious adverse event
SOP	Standard operating procedure
SSP	Study specific procedure
Temp	Temperature
TLR 9	Toll-like receptor 9
USAMMDA	U.S. Army Medical Materiel Development Activity
USAMRMC ORP HRPO	U.S. Army Medical Research and Materiel Command, Office for Research Protection, Humans Research Protection Office
VS	Vital signs
WRAIR	Walter Reed Army Institute of Research

IV. BACKGROUND INFORMATION

IV.1. Introduction

For more than a century, humans have been afflicted with illnesses characteristic of influenza virus infection. Influenza virus is a respiratory pathogen belonging to the family of *Orthomyxoviridae* that causes annual epidemics and occasional pandemics {Cox, 2004}. In a pandemic, global spread of a new influenza virus, unrelated to viruses circulating prior to the outbreak, results in increased morbidity and mortality. Recently, several avian viruses have crossed the species barrier and directly infected humans, presenting a possible pandemic threat. One of these is avian influenza H5N1 virus, with a mortality rate of more than 50% {Beigel, 2005}. The first documented cases of human infection with avian influenza H5N1 virus occurred in Hong Kong in 1997, when 18 people were reported to be infected, among whom 6 died{Claas, 1998}. So far, H5N1 viruses are primarily animal pathogens and mainly circulate in poultry. Most human cases to date have arisen from close contact to infected poultry or their waste products; human-to-human transmission evidently remains very limited {Fauci, 2006}. Even so, the high case-fatality rate in humans and the concern that mutant viruses might acquire the capacity for efficient transmission among humans has caused great concern worldwide.

A major strategy to combat pandemic influenza is the generation of an effective vaccine that induces protective immune responses through neutralizing antibodies {Katz, 2006 #34}. Several H5N1-specific vaccines are currently being developed; however, high containment conditions are required to grow and purify highly pathogenic H5 vaccines and in addition chicken egg-based production is limited because H5N1 is lethal to eggs. Also, since influenza viruses undergo unpredictable antigenic shifts, effective vaccines should induce broad immune responses that are not limited to one strain of H5N1.

Live, attenuated influenza vaccine (LAIV) is an intranasally administered vaccine, designed to induce an immune response resembling infection with wild-type influenza without causing disease{Cox, 2004}. Compared to conventional intramuscular inactivated vaccines, LAIV induces higher amounts of mucosal antibodies and broader immune responses, including cellular immunity. Even, LAIV can induce responses to antigenically mismatched influenza A strains. We hypothesize that heterologous prime-boost immunization of humans with LAIV and inactivated influenza vaccine will induce broader immune responses and cross-immunity to a variety of H5N1 avian influenza viruses.

IV.2. Immunology of Influenza and Influenza Vaccines

IV.2.1. Influenza Virus

Influenza virus is a globally occurring respiratory pathogen which causes a high degree of morbidity and mortality every year. There are three types of influenza virus, A, B and C, which differ in their internal proteins, nucleoprotein and matrix protein. Type A is found in a wide variety of warm blooded animals, including birds and human, whereas B and C are restricted to human. Disease is mainly caused by influenza A and B. Influenza A can be classified into subtypes on the basis of the surface glycoproteins hemagglutinin (HA) and

neuraminidase (NA), of which 16 (H1-H16) and 9 (N1-N9) subtypes exist, respectively. All subtypes have been found in aquatic ducks, but only some can infect humans.

Influenza is continuously undergoing changes in its surface glycoproteins to escape host immunity. There are two mechanisms for this continual emergence of variants, antigenic drift and antigenic shift. Antigenic drift is caused by an accumulation of point mutations in HA and NA genes, resulting in minor changes in amino acids that allows the virus to escape neutralizing antibodies from previous infections or immunizations. Antigenic drift leads to new epidemic strains; this phenomenon drives the annual reformulation of the influenza vaccine. Antigenic shift occurs only rarely when a novel HA derived from avian of animal origin appears in humans. If this novel virus is capable of spreading from human-to-human, it may cause a pandemic.

Influenza viruses grow rapidly in human respiratory mucosa, allowing human-to-human transmission via respiratory virus-laden droplets (coughs and sneezes) even before onset of symptoms. In this way, influenza outbreaks can rapidly move through a population. The symptoms are respiratory, including cough, sore throat, rhinitis, as well as systemic, such as fever, myalgia, headache and severe malaise. Uncomplicated influenza resolves itself within 1 to 2 weeks; however, in persons with underlying medical conditions, young infants and elderly people, influenza can lead to hospitalization and even death.

IV.2.2. Avian influenza H5N1

The first documented cases of human infection with avian influenza H5N1 virus occurred in Hong Kong in 1997, when 18 people were reported to be infected, among whom 6 died{Claas, 1998}. In Asia, wild and domestic aquatic birds provide the natural reservoir for H5 influenza viruses; live-poultry markets in Hong Kong were found to be the source of the virus transmitted to humans {Webster, 2006}. Although the H5N1 viruses remained relatively unchanged from 1997 to 2002, in later years marked antigenic drift occurred resulting in H5N1 highly pathogenic to chickens and wild birds; of note, certain wild birds show no disease signs but are a source of infectious H5N1. This went hand in hand with spreading of H5N1 across Asia and later Europe and Africa, through migratory birds or transport of live birds. At the same time, additional cases of avian-to-human transmission of H5N1 were reported in Vietnam, Thailand, Cambodia and Indonesia, as well as some African countries.

To date (28 May 2008), 383 cases have been reported of which 241 resulted in death (www.who.int). These represent only laboratory-confirmed cases of H5N1 infection and actual numbers might be higher. Still, the number of human cases is limited if the continued high circulation in wild and domestic birds is taken into account. Human-to-human transmission was probably only involved in a minority of cases. Even so, pandemic preparedness is necessary, since there is no doubt that an influenza pandemic will occur and rapidly changing H5N1 viruses are prime candidates to cause this pandemic{Webster, 2006}. Of great concern is the potential of reassortment of H5N1 with human viruses and/or adaptation of H5N1 to the human host that would result in a pandemic strain that can be transmitted from human-to-human. Combined with the high mortality of H5N1 (61%), this could cause a global catastrophe.

IV.2.3. Immune responses to influenza virus

The immune system recognizes and fights any incoming pathogen. It consists of two parts, the humoral immune system responsible for antibody production and the cellular immune system, including cytotoxic T lymphocytes that kill infected cells. For the resistance to and recovery from influenza, antibodies are the most important{Cox, 2004}. During the course of infection, antibodies are produced against a variety of influenza antigens, of which HA-specific antibodies are the most effective for neutralization of the virus and thus prevention of illness. However, antibodies directed against other influenza proteins can limit viral replication and ameliorate disease. After natural infection, a protective serum antibody response (haemagglutination inhibition titre $\geq$40) can be detected in about 80% of subjects and the level of serum antibodies correlates with resistance to illness following natural and experimental infection{Cox, 2004}. The major neutralizing antibodies are of the IgA class, but also IgM and IgG antibodies are detected after infection. Locally, in mucosal tissues, IgA is most prominent, whereas in serum IgG levels are dominant. Antibody levels peak after 2 weeks and then begin to decline.

The cellular immune system plays a role in recovery from influenza infection and prevention of influenza-associated complications {Doherty, 2006}{Cox, 2004}. Cytotoxic T-lymphocytes (CTLs, also called $CD8^+$ T cells) can lyse influenza infected cells in concert with influenza-specific antibodies and complement. CTL levels peak 2 weeks after infection and return to baseline after 6 months. Interestingly, influenza-specific CTLs exhibit cross-reactivity between influenza A strains, *i.e.* they lyse cells infected with other influenza A subtypes. In addition to $CD8^+$ T cells, influenza-specific $CD4^+$ T cells are also induced during infection. These so-called T-helper cells mainly function to promote high-quality antibody responses.

IV.2.4. Influenza vaccines

The primary method for prevention of influenza and its associated complications is vaccination. The World Health Organization has a global influenza surveillance network to detect circulating strains; based on this information they determine which strains are used in all vaccines to match the circulating strains. Current vaccines are trivalent and contain two subtypes of influenza A and one subtype of influenza B (season 2007/2008: A/Solomon Islands/3/2006 (H1N1)-like virus; A/Wisconsin/67/2005 (H3N2)-like virus; B/Malaysia/2506/2004-like virus). The viruses for vaccine are propagated in embryonated hens' eggs and subsequently purified; this whole process requires at least six months. Two general types of vaccines are currently licensed for use: inactivated influenza vaccines (IIVs) and live, attenuated influenza vaccines (LAIVs). For IIVs, that are licensed worldwide, viruses are inactivated with formaldehyde or beta-propiolactone. IIVs are available as whole or subunit (purified surface glycoproteins) formulations and are administered intramuscularly or subcutaneously {Katz, 2006}. After administration of IIV, the level of serum anti-HA antibodies correlates with protection against influenza infection. Recent work has also demonstrated step-wise increasing antibody responses to multiple doses of inactivated influenza vaccine (A/H1N1) in healthy human volunteers that are not specific for just the vaccine strain of virus but also to antigenically different influenza viruses { Keitel 2008}.

IV.2.5. Live, attenuated influenza vaccines

The goal of LAIVs is to induce an immune response that more closely resembles that of natural infection. LAIVs are delivered intranasally and replicate to a limited extent in the upper respiratory tract. In this way, they induce immune responses similar to those induced by natural infection. The viruses used are based on the concept of cold-adaptation resulting in attenuated influenza master strains that grow at 33°C (as in nasal passages, which are below body temperature) but not at higher temperatures. These master strains can subsequently be recombined with the HA and NA genes of the annually recommended vaccine strains, resulting in reassortants that possess the attenuated phenotype of the master strain and the desired antigenic properties of the epidemic strains. LAIVs are currently only licensed for use the USA (FluMist®) and in Russia.

Compared to IIVs, LAIVs induce a similar frequency of systemic vaccine reactions and a similar efficacy in preventing influenza {Beyer, 2002}. Serum levels of anti-influenza antibodies were lower, whereas mucosal IgA levels were higher as compared to IIVs. Unlike IIV, no general immune correlates of protection have been established for LAIV{Ambrose, 2006}. As observed for natural infections, not all differences in protection could be accounted for by differences in serum antibody titers indicating that multiple immune mechanisms confer resistance to influenza. The presence of an antibody response is predictive of protection; however, the absence of antibodies does not reflect the absence of protection. Of note, LAIVs induce broader antibody responses, not only to HA proteins but also to other influenza proteins {Ambrose, 2006 38}{Belshe, 2004}. Even, responses are not limited to the strains used for vaccination, but neutralizing antibodies are also induced that cross-react with mismatched strains. Five different efficacy trials have demonstrated that immunization with LAIV can protect against antigenically drifted influenza strains, in addition to providing protection against the influenza strains used in the vaccine{Belshe, 2004}. This was confirmed in vitro, by testing sera on antigenically mismatched strains; vaccination with LAIV resulted in antibodies to drifted variants whereas IIV could not induce such cross-reactive antibodies. These results were obtained using antigenically drifted influenza strains within the same subtype (variants of H3N2); no results are available for antigenically shifted strains (e.g. H5N1). In contrast to IIV, LAIV not only generates antibody responses, but also cytotoxic T cell responses.

IV.2.6. Vaccines against avian influenza H5N1

In the event of a flu pandemic, it is unlikely that production of a new vaccine using the classical method of embryonated hens' eggs is fast enough to meet expected demand. For avian influenza viruses, there are several more problems for the generation of vaccine candidates. Avian influenza viruses are lethal to embryonated eggs, which limits growth in large quantities. In addition, high containment conditions are required to grow and purify highly pathogenic H5 vaccines. Also, it might be too risky to give a live, attenuated variant of H5N1 to humans. Since influenza A viruses undergo unpredictable antigenic shifts, effective vaccines should induce broad immune responses that are not limited to one strain of H5N1.

Despite these obstacles, several candidate H5N1 vaccines have been tested in clinical trials, all based on inactivated vaccines{Treanor, 2006} {Treanor, 2001}{Bresson, 2006}{Lin, 2006}{Leroux-Roels, 2007}{Ehrlich 2008}. All vaccines required a two-dose strategy to

raise high antibody titers and cross-reactivity to heterologous H5N1 strains, even in the presence of adjuvant {Subbarao, 2007}. An alternative strategy is the use of non-pathogenic H5 strains to induce cross-reactivity to highly pathogenic strains. Using an H5N3 surface-antigen vaccine (IIV), Stephenson et al showed that cross-reacting antibody responses to H5N1 could be induced, but only after 2 doses with addition of an adjuvant or after 3 doses of a nonadjuvanted vaccine{Stephenson, 2005}{Stephenson, 2003}. Similarly in mice, an adjuvanted non-pathogenic H5N2 IV could generate cross-protection against challenge with highly pathogenic H5N1 {Lu, 2006}. Interestingly, in the same study, one dose of non-pathogenic H5N2 LAIV gave similar levels of protection against H5N1 as compared to two doses of IIV.

Yet another approach would be to use currently licensed seasonal trivalent influenza vaccines to induce cross-protection against H5N1. This has only been tested in mice, using 3 doses of adjuvanted 2005-2006 seasonal IIV (containing H1N1 and H3N2){Ichinohe, 2007}. The observed cross-protection against H5N1 challenge in this study was likely mediated by mucosal IgA specific to viral internal protein, obtained upon intranasal inoculation, but not when using the subcutaneous route. The use of licensed seasonal trivalent influenza vaccines to elicit cross-immunity against H5N1 is further supported by recent published data showing that antibodies against neuraminidase (N1), and viral conserved internal proteins (matrix protein and nuclear protein) were able to induce cross-protection in mice against H5N1 references {Sandbulte, 2007} {Huleatt, 2008} {Lalor, 2008} {Ernst, 2008}.

In general, immune responses to vaccines can be increased by 1 of 3 ways: addition of adjuvant and/or administration of multiple doses and/or a combination of 2 different vaccine strategies (heterologous prime-boost). Since H5N1 is antigenically diverse from current human influenza, it is expected that a different strategy will be needed to induce protective immune responses as compared to seasonal influenza. Indeed, multiple doses were required for all candidate H5N1 vaccines or vaccines that induce cross-protection tested so far and most candidate H5N1 vaccines were poorly immunogenic without adjuvant (Subbarao, 2007). Alternatively or additionally, two diverse vaccines could be used sequentially in a so-called heterologous prime-boost strategy to increase and broaden immune responses. It has been well documented that heterologous prime boost vaccination elicits high-magnitide, broad-based and long-lasting immunity in several different animal and disease models {Verrier, 200} {McConkey,2003} {Gomez, 2007}. In such a prime-boost vaccination, one vaccine is used to initiate immune responses (“prime”), followed by a second immunization with a different vaccine to boost immune responses. In the case of influenza, LAIV and IIV could be combined as a prime-boost vaccination. This approach has several advantages over using two immunizations with the same vaccine. Firstly, upon a 2nd immunization with LAIV memory responses will rapidly lead to elimination of the virus, thus drastically reducing viral replication, immune responses and ultimately vaccine efficacy. More importantly, we expect that by combining a mucosal vaccine containing live virus with an intramuscular vaccination using inactivated virus, induced immune responses will be broader and higher, both systemically and mucosally. Our approach, therefore, is to compare cross-reactivity of two doses of seasonal LAIV or IIV to H5N1 versus a prime/boost dosing of LAIV followed by IIV or vice versa. We hypothesize that such a

prime-boost strategy will favor generation of both IgA and IgG antibodies that are able to cross-react with and protect against H5N1.

IV.3. FluMist® Live Attenuated Influenza Vaccine

IV.3.1. Product information

FluMist® was first approved by the FDA in 2003 for the prevention of disease caused by influenza A and B viruses in healthy subjects aged 5 to 49 years. Even though it was considered safe in the 50-64 year old age group the data on efficacy was not considered strong enough for licensure to be applied to this group. Following clinical trials the indication for use was broadened to include children aged 2 years and older. Initially FluMist® was a frozen formulation; however in 2007 the formulation was converted to a refrigerated liquid, which requires storage at 2-8ºC. FluMist® is licensed for use only in the USA.

FluMist® is a live, trivalent vaccine for administration by intranasal spray. The influenza virus strains are (a) cold-adapted, meaning they replicate well at 25°C, a temperature that is restrictive for replication of many wild-type influenza viruses; (b) temperature-sensitive, meaning they are restricted in replication at 37°C (Type B strains) or 39°C (Type A strains), temperatures at which many wild-type influenza viruses grow efficiently; and (c) attenuated, meaning they do not produce classic influenza-like illness in the ferret model of human influenza infection. The combined effect of the antigenic and phenotypic properties is that the attenuated viruses replicate in the nasopharynx to induce protective immunity.

For each of the 3 reassortment strains in FluMist®, 6 internal gene segments responsible for (a) to (c) phenotypes are derived from a master donor virus (MDV), and the 2 segments that encode HA and NA are derived from the corresponding antigenically relevant wild-type influenza viruses that have been recommended for inclusion in the annual vaccine formulation. Thus the 3 viruses contained in FluMist® maintain the replication characteristics and phenotypic properties of the MDV and express the HA and NA of wild-type viruses that are related to strains expected to circulate during the 2008-2009 influenza season.

Each of the reassortment strains is inoculated into specific pathogen-free eggs and incubated to allow viral replication. The monovalent bulk is then harvested, filtered and combined to form trivalent vaccine. Each pre-filled sprayer contains a single 0.2ml dose. Each dose contains 10FFU (fluorescent focus units) of live attenuated influenza virus reassortments of each of the 3 strains (for the 2008/9 Northern Hemisphere season these will be: an A/Brisbane/59/2007 (H1N1)-like virus, an A/Brisbane/10/2007 (H3N2)-like virus, and a B/Florida/4/2006-like virus), monosodium glutamate, hydrolyzed porcine gelatin, arginine, sucrose, dibasic potassium phosphate, monosodium phosphate, and <0.015mcg gentamicin sulphate. There are no preservatives.

IV.3.2. Safety and tolerability following single and dual dose administration in healthy subjects

Pre-licensure clinical trials, in which more than 20,000 subjects (of whom 3,700 were healthy adults) received more than 28,000 doses of LAIV (FluMist®), showed the vaccine to be safe and well-tolerated (Belshe 2004). In the largest published study of safety in healthy adult volunteers, a randomized double-blind, placebo-controlled trial of LAIV, the following AEs were reported following a single dose of vaccine: runny nose 44%; sore throat 27%; fever 1%; cough 17%; headache 40%; muscle aches 19%; tiredness 23%. Most of these AEs were reported equally between the LAIV and placebo groups; however runny nose and sore throat were significantly more likely to be reported in the LAIV group (17% and 10% increase respectively) (Nichol 1999). Most vaccine-related AEs lasted just 1-2 days and occurred during the first 7 days after vaccination. There was no increase in use of concomitant medications such as antibiotics or cold remedies in LAIV recipients. There were no SAEs attributable to LAIV.

Post-licensure AEs in FluMist® recipients reported to VAERS (US Vaccine Adverse Event Reporting System) during the first 2 seasons of FluMist® use were published in 2005 (Izurieta 2005). There were no unexpected serious risks identified. Out of approximately 2.5 million recipients, 460 AE reports were received: 217 involved respiratory events (67 were influenza-like and 2 of these were serious influenza-like illness); 7 possible cases of anaphylaxis of which 5 were within 20 minutes of vaccination (giving an anaphylaxis rate of 2 per million vaccines which is similar to that of MMR); 47 other allergic; 2 Guillain Barre syndrome; 1 Bells palsy; 8 asthma exacerbation in individuals with a prior asthma history; 13 epistaxis; 33 abdominal symptoms; 67 constitutional symptoms (21 weakness/tiredness; 14 fever; 13 headache; 12 dizziness; 7 arthritis). There were no deaths associated with vaccination.

In children, the AE profile seems similar to that in adults. Two studies reported 11% more nasal congestion/rhinorrhea in 1038 vaccinated children aged 15 to 71 months, a greater use of analgesics and antipyretics after the first dose and a non-significant trend towards more fever and reduced activity levels (4% in both instances) in the vaccine groups compared to placebo recipients (Belshe 1998, Piedra 2002). These differences were only observed on the 2nd and 3rd days following vaccination, although these symptoms were also evident again on days 8 and 9. The odds ratios of developing significant symptoms compared to placebo up to 10 days after vaccination were as follows: runny nose/nasal congestion 1.61; vomiting 1.78; muscle ache 2.0; fever 1.52. Other reported symptoms did not occur at an increased rate after controlling for variables such as age and child care attendance. Again there were no associations between vaccine use and SAEs, otitis media or use of medications. There was a 21% reduction in the overall incidence of febrile illnesses during 8 months after the start of the study.

There are no published studies detailing safety in healthy adults following dual dosing with FluMist®. There are some published reports of safety following intranasal vaccination with precursors of FluMist, in which no increase in AEs were seen following the second dose, though reduced viral shedding was observed (Kietel 1993, Reeve 1980). However 2 doses of FluMist® one month apart is the licensed vaccine regimen for previously unvaccinated

children aged 2-8 years because of the improved immune response following the second dose (Belshe 1998, 2004, 2007, Tam 2005). In children the second dose appears to be better tolerated than the first with no excess of AEs compared to a placebo comparator group. It is reasonable to suppose that the AE profile in adults would be comparable to that in children and that a second dose of FluMist® would be safe and well-tolerated.

IV.3.3. Safety in special groups

Because FluMist® is a live vaccine, immunocompromised subjects and those with chronic medical conditions are currently advised to receive inactivated vaccine rather than FluMist®. However because of the advantages conferred by inducing a broad cross-protection with LAIV compared to IIV, and because of the theoretical concerns that viral shedding from vaccine recipients may cause secondary transmission of live vaccine strains to vulnerable groups, a number of small studies has been conducted to try and address the issue of whether or not LAIV is safe in certain special groups:

- Pre-school children. Since the main mode of transmission of influenza appears to be via droplets from young children, immunizing this group was seen as an important step in limiting influenza spread within the household. However one study of LAIV in young children (Bergen 2004), a DBRPC study of FluMist® versus placebo in which 9689 children aged 12 months to 17 years were enrolled, found a statistically significant increase in risk of asthma/reactive airways disease in children aged 12 to 36 months following administration of FluMist® (relative risk 4.1 (90% CI 1.29 to 17.86)). This finding has not been repeated in other studies with similar design (Ashkenazi 2006, Fleming 2006, Piedra 2005). However, a further study by MedImmune (Package insert, Appendix 1) showed that wheezing was more common in FluMist® recipients aged 6-23 months than active controls (5.9 and 3.8% respectively), though not in children aged 24 to 59 months, while rates of all-cause hospitalization were also more frequent in the 6 to 23 month age group. A recent study in babies less than 6 months old showed more irritability and nasal congestion in those infants aged 6 to 15 weeks compared to a placebo group but no other clinical signs (Vesikari 2008). The effects were seen after Dose 1 but not after Dose 2. For these reasons FluMist® is currently not recommended for use in children aged less than 2 years.

- Asthma. In children aged 9 to17 years with a diagnosis of moderate to severe asthma there was no effect of LAIV on asthma and no differences in post-vaccination symptoms between vaccinated and placebo groups (Redding 2002).

- HIV-positive subjects. LAIV was well-tolerated by 26 HIV positive adult subjects with relatively good health in a DBRPC trial (King 2000). There was no increased risk of AEs in the HIV positive recipients compared to HIV negative recipients, no effect on HIV viral load, CD4 counts and no prolongation of viral shedding post vaccination. LAIV recipients were more likely to have nasal congestion than placebo recipients regardless of HIV status. Similar results were obtained from a study in HIV-positive and negative children in which 2 doses were given 1 month apart in a RDBPC trial (King 2001). Both studies concluded that inadvertent vaccination with LAIV in

relatively asymptomatic HIV-infected individuals would not be associated with frequent significant AEs.

- Elderly subjects with chronic medical conditions. LAIV or placebo was given intranasally along with intramuscular IIV to 200 subjects aged 65 years or older who had a chronic medical condition (cardiovascular disease 65, pulmonary disease 11, diabetes mellitus 53) (Jackson 1999). Other than sore throat, which was significantly more frequently reported in LAIV than placebo recipients (15 versus 2%), the vaccine was safe and well-tolerated in this heterogeneous group of subjects.

- Pregnancy and lactation. There have been no detailed animal reproduction studies involving FluMist®, hence it is not known whether it can harm the fetus or affect reproduction capacity. In a single unpublished study of rats administered vaccine once or twice before gestation, during organogenesis or both, large doses of vaccine had no effect on pregnancy, parturition, lactation, embro-fetal or pre-weaning development, and no evidence of teratogenesis (Product Prescribing Information, Appendix 1a). It is not known if FluMist® is excreted in human milk. Although not specifically contraindicated the manufacturer advises against use of FluMist® in pregnant and nursing women.

IV.3.4. Secondary transmission of vaccine virus

Because FluMist® contains live, attenuated influenza viruses that must infect and replicate in cells lining the nasopharynx of the recipient to induce immunity, vaccine viruses capable of infection can be cultured afterwards from nasal secretions. A small study in healthy adults showed that 50% of subjects shed vaccine virus on Day 3 after vaccination, but none shed between Days 10 and 21 (Talbot 2005). In a RDBPC study in a day-care setting in children aged less than 3 years at least 1 vaccine strain was isolated from 80% of recipients during 21 days post-vaccination (Vesikari 2006). Transmission of at least 1 vaccine strain to non-vaccinated close contacts within the centre was calculated to be 2.4% (95%CI 0.13 to 4.6), while the probability of transmission to a child after contact with a single vaccinated child was 0.58% (95%CI 0-1.7). Duration of vaccine shedding has not been definitively established, though in the above study the mean duration was 7.6 days ± 3.4 days.

Post-licensure AEs in 2.5 million FluMist® recipients reported to VAERS during the first 2 seasons of FluMist® (Izurieta 2005) included 22 reports of possible secondary transmission of the vaccine virus from vaccinees to non-vaccinees. None of these events resulted in hospitalization. Ages ranged from 3 to 64 years. The interval from exposure to a vaccinated person to symptom-onset ranged from a few hours to 15 days. 59% of the events were transmission to a health care clinician who administered the vaccine, although almost half of the vaccinees had respiratory symptoms at the time of vaccination. Secondary transmission was not confirmed by laboratory tests in any of the cases. There have been no reports of secondary transmission to an immunosuppressed individual.

IV.4. Fluzone® Inactivated Influenza Vaccine

IV.4.1. Product Information

Fluzone® is a formaldehyde-inactivated vaccine made from influenza viruses propagated in embryonated chicken eggs and designed for intramuscular use. The virus is chemically disrupted using a surfactant and then further purified and suspended in buffered isotonic sodium chloride solution. It is then standardized according to USPHS requirements for the influenza season and formulated to contain 45 micrograms HA per 0.5ml dose in the same recommended ratio as for FluMist® (Section IV.3.1). Gelatin is used as a stabilizer. It is supplied both with a preservative (thiomersal, a mercury derivative) and also without any preservative, as a 0.5ml pre-filled syringe or vial. No antibiotics or latex are used in the manufacturing process. The dose for adults is 0.5ml.

IV.4.2. Safety and tolerability following single and dual dose administration in healthy subjects

Because this vaccine contains killed viruses only it cannot cause influenza-like symptoms or viral shedding. Following single dose administration local reactions in placebo-controlled studies in adults consist mainly of soreness at the vaccination site (10-64% of subjects) lasting up to 2 days, local pain and swelling.These local reactions typically are mild and rarely interfere with the persons abilities to conduct usual daily activities. Systemic reactions, such as fever, malaise, myalgia and headache, are uncommon in healthy adults and older persons when compared with placebo injections. Systemic reactions that do occur begin 6-12 hours after vaccination and can persist for 1-2 days. Young children who have had no prior exposure to influenza antigens in the vaccine are more likely to experience systemic symptoms. Immediate allergic reactions, such as hives, angioedema, allergic asthma and systemic anaphylaxis occur extremely rarely and are believed to result from hypersensitivity to certain vaccine components, the majority probably due to egg protein.

Guillain Barre Syndrome has been reported following influenza vaccination although the association remains unclear; for every 1 million persons vaccinated there is approximately 1 additional case of GBS (Haber 2008). Potential vaccinnees who have previously had GBS may be at increased risk of GBS following influenza vaccination. There have also been rare reports of neurological disorders such as encephalopathy, facial paralysis, optic neuritis and brachial plexus neuropathy in the 2 weeks following vaccination; full recovery was almost always reported (Schonberger 1979, Retaillaiu 1980, Guerrero 1979).

As for FluMist®, there is only very limited information about safety and tolerability following a second dose of IIV, and there is no information about a second dose of Fluzone® specifically. Two doses of trivalent IIV (such as Fluzone®) one month apart is the licensed dose for previously unvaccinated children aged less than 9 years. While there are no published studies in healthy adults, Englund and colleagues gave 2 or 3 doses of IIV to 259 6-23 month old children over an 8-month period, of which the last two doses were separated by 30 days; There was no increase in the incidence of fever, pain, redness or

swelling at the vaccination site following the 2nd or 3rd doses, suggesting that additional doses of IIV are well-tolerated (Englund 2005). However a study by Neuzil et al in 5-8 year old children given 2 doses of IIV (Sanofi Pasteur 2004-5 vaccine) 4 weeks apart showed more frequent reports of pain following the second dose (71 versus 59% after the first dose) but increased reports of redness, swelling or itching at the injection site after dose 2 (Neuzil 2006). A high pre-vaccination antibody titer ($\geq$1:10) of B/Jilin/20/2003 compared with a B/Jilin/20/2003 titer of <1:10, but not other anti-influenza antibodies, was found to be predictive of moderate/severe pain after dose 2 (relative risk 1.60, 95% CI 1.01-2.56). Thus it is possible that some subjects could experience more pain or discomfort at the injection site following a second dose of intramuscular IIV than is experienced following the first dose.

In adults there are some data on dual dosing in subjects believed to be at risk of mounting a poorer immune response to influenza vaccine than healthy adults. However these reports focus more on vaccine efficacy rather than detailed safety or tolerability. In a non-randomized study of 2 doses of IIV given 1 month apart to 109 subjects aged 33 to 41 years (31 HIV-seronegative controls, 32 asymptomatic HIV seropositive, and 46 HIV-seropositive with AIDS), there was no reported detrimental effect following administration of the second dose (Miotti 1989). Of note, although the subjects with symptomatic HIV did not have an improved level of immune response following the second dose of vaccine, a greater proportion of the uninfected control group did attain protective immunity following the second dose (73 and 82%, 100 and 100%, and 45 and 64% for the 3 vaccine strains of influenza after doses 1 and 2 respectively). Two studies in adult hemodialysis patients using 2003/4 vaccine also failed to demonstrate additional protection from a second dose of IIV given 1 month after the first, but did not report any safety or tolerability concerns following the 2nd dose (Tanzi 2007; Song 2006).

IV.4.3. Safety in special groups

- Elderly subjects with chronic medical conditions. Fluzone® plus placebo was given intranasally along with intramuscular Fluzone® to 200 subjects aged 65 years or older who had a chronic medical condition (cardiovascular disease 65, pulmonary disease 11, diabetes mellitus 53) (Jackson 1999). The vaccine was safe and well-tolerated in this heterogeneous group of subjects. AEs are described in Section IV.4 below.
- Pregnancy and lactation. The vaccine is safe during pregnancy and lactation and is recommended in the US for all women who will be pregnant during the influenza season.
- HIV-infected individuals. Fluzone® is safe when administered to HIV-infected individuals.
- Asthma: Fluzone® is safe and is recommended for sufferers of this condition
- Pre-school children: Fluzone® is safe and is specifically recommended for children aged 6-23 months

IV.5. Concomitant Administration of FluMist® and Fluzone®

If Flumist® is administered at the same time as IIV the 2 vaccines seem to be well-tolerated. Jackson et al (Jackson 1999) studied 200 seniors with chronic medical conditions such as coronary artery disease, previous myocardial infarction, COAD, emphysema or diabetes. In a RDBPC study all subjects received IIV (Fluzone®in all but 2 cases) followed straight away by intranasal LAIV or placebo. The IIV plus LAIV group reported occurrence of sore throat significantly more frequently than the placebo group (15 vs 2%). There were no differences between the groups for other reported AEs: pain at injection site (16 and 15%), redness at injection site (8 vs 9%), fever (3 vs 1%), cough (10 vs 9%), runny nose (28 vs 24%), headache (16 vs 15%), chills (5 vs 7%), muscle aches (15 vs 14%) or tiredness (20 vs 16%). There were 2 SAEs in each group, which were unrelated to the study, and no deaths

IV.6. Study Justification

Both LAIV and IIV contain neuraminidase (N1) which is the same subtype of neuraminidase found in avian influenza H5N1. In addition other viral proteins in human influenza vaccines (LAIV, IIV), including matrix protein 2 and nucleoproteins, are highly conserved among influenza A virus. Recently published work has shown that the immune responses against these viral proteins will very likely be cross-reactive to H5N1 (Ichinohe, 2007; Keitel, 2008).

Because of the difficulties encountered in producing a specific H5N1 vaccine, and the animal data showing appreciable cross-protection of LAIV with other influenza A viruses, this study will contribute significantly to our understanding of human immune cross-protection to H5N1 following single, dual or prime boost vaccination with currently available seasonal influenza vaccines. The dosing regimes proposed here for healthy adults are designed to achieve the maximal immune response so that the chance of detecting any measurable cross-protection is maximized. Although safety data on dual dosing with influenza vaccine in healthy adults is limited, the available data from children and adults with chronic medical conditions do not suggest there will be significant additional risks from the second dose.

IV.7. Additional Background Information and Justification for Amendment 6

Memory B cells are known to persist for a lifetime and therefore represent an ideal repository for antibody specificities generated by an individual after vaccination (Lanzavecchia, 2009). Unlike plasma cells, memory B cells can be immortalized by Epstein Barr virus (EBV). The EBV-transformed memory B cells produce large amount of antibodies; however the efficiency of B cell immortalization and cloning is low. An improved method of EBV immortalization has now been established using the TLR9 ligand CpG ODN, which is able to enhance the efficiency of EBV B-cell immortalization more than 100-fold (Traggiai, 2004). This method has been employed to dissect the human antibody response to pathogens such as, SARS (Traggiai, 2004), avian influenza H5N1 (Simmons, 2007), HIV (Corti, 2010), and dengue (Beltramello, 2010).

Preliminary analysis of data from WRAIR #1525 indicates that serum from a subset of subjects exhibited potent *in vitro* cross-reactivity to H5N1 and pandemic H1N1 2009. The observed antibodies are probably generated from long-lived, cross-reactive memory B cells produced in response to a prime-boost vaccination with seasonal influenza vaccine, since it is likely that these subjects had never previously been exposed to H5N1 and/or pandemic H1N1 2009.

We propose to attempt to produce human monoclonal antibodies from the original samples collected from those subjects with high responses to allow for further analysis of the immune response to influenza vaccines. Subjects will be re-contacted to assess willingness to participate in an additional blood sampling under a minimal-risk human use protocol. A greater understanding of cross-reactive memory B cell responses to influenza neuraminidase and hemagglutinin may eventually lead to new/novel preventive and therapeutic strategies.

V. OBJECTIVES

The hypothesis to be tested in this study is that heterologous prime-boost vaccination of healthy adults with a prime boost regimen of trivalent LAIV (FluMist®) and trivalent IIV (Fluzone®) will induce cross-immunity to a variety of H5N1 avian influenza viruses and human influenza viruses.

Therefore the primary objectives of this study are:

1. To establish whether a prime boost strategy using seasonal LAIV and IIV can augment cross-reactivity with H5N1 and human influenza strains.
2. To detect the presence of antibodies that can inhibit infection with H5N1 and human influenza strains in microneutralization, plaque reduction, or H5 pseudotyped lentiviral particle-based assays after vaccination with seasonal LAIV and IIV.
3. To establish the breadth of antibody responses to H5N1 strains and human influenza strains using ELISA, microneutralization, plaque reduction, H5 pseudotyped lentiviral particle-based assays, neuraminidase inhibition assay, and hemagglutination inhibition after vaccination with seasonal LAIV and IIV.
4. To observe T-cell responses following vaccination with seasonal LAIV and IIV.
5. To attempt to generate human monoclonal antibodies that are cross-reactive to enzyme neuraminidase and hemagglutinin of H5N1 and pandemic H1N1 2009 in order to investigate further the human response to influenza vaccination

Secondary objectives are to describe the adverse event profile of 2 doses of influenza vaccine given approximately 8 weeks apart in healthy adult volunteers.

VI. METHODS

VI.1. Study Endpoints

The primary study endpoints will be determination of pre and post vaccination immune responses in 26 healthy human subjects to 2 doses of seasonal influenza vaccine including whether there is *in vitro* cross-reactivity against H5N1 virus as follows:

1. Antibodies ELISA titers calculated as the dilution factor required to give an optical density of 0.5.

2. Hemagglutination inhibition activity defined as the titer required to completely inhibit hemagglutination);

3. Microneutralization assay endpoint titer; determined by using 50% specific signal calculation. The endpoint titer will be expressed as the reciprocal of the highest dilution of serum with A490 value less than X, where X = [(average A490 of VC wells) – (average A490 of CC wells)] / 2 + (average A490 of CC wells).

4. Neuraminidase inhibition assay titer defined as the highest sample dilution that produces 50% inhibition of virus control.

5. Plaque reduction assay titer assessed as the highest sample dilution that will give a reduction of plaque count of 50%.

6. H5 pseudotyped lentiviral particle-based serological assay titer assessed as the highest sample dilution that produces 50% inhibition of virus control.

7. Influenza specific T-cell cytokine production by influenza-specific T-cells defined as the the percentage of cytokine-staining cells at least twice background.

8. Antibody staining against matrix 2 protein (M2e) expressed cell line. The samples considered positive are those in which the staining intensity is at least twice that for the background.

9. Human monoclonal antibody production from peripheral blood memory B cells isolated from selected subjects

VI.2. Study Design

This will be a randomized, open-label, pilot feasibility study of four 2-dose vaccine regimens using two commercially available seasonal influenza vaccines. The *in vivo* phase of the study will entail randomization of up to 26 adult volunteers to receive: either 2 doses of LAIV (5 subjects) (Autologous prime boost regimen 1), 2 doses of IIV (5 subjects) (Autologous prime boost regimen 2), one dose of LAIV followed by one dose of IIV (8 subjects) (Heterologous prime boost regimen 1) or one dose of IIV followed by one dose of LAIV (8 subjects) (Heterologous prime boost regimen 2) (Appendix 2). AE data will be collected into a symptom diary for 14 days following each dose of vaccine. Serum and local (nasal) immunological responses will be evaluated 2 and 4 weeks after each dose. A further 8 subjects will be recruited to act as replacements in case of drop-outs.

VI.3. Study Site

The *in vivo* phase of the study will take place at the U.S. Embassy Medical Unit, Bangkok, Thailand. In selected cases individuals may elect to have scheduled follow-up visits (which do not involve vaccination) performed at AFRIMS. Immunology testing will be done at the

Department of Immunology and Medicine, AFRIMS and the Faculty of Medicine, Siriraj Hospital, Bangkok.

VI.4. Population
Subjects will be healthy U.S. citizens residing in Bangkok who fulfill the inclusion criteria (Section VII.1) and do not meet any exclusion criteria (Section VII.2). FluMist® vaccine is not yet licensed for use in Thailand; hence Thai subjects will not be recruited into this study.

VI.5. Recruitment
Potential subjects will be recruited by the use of flyers posted in several locations around Bangkok where US citizens are known to frequent, for example at the US Embassy. Interested individuals will be invited to telephone a study number where they will be able to ask questions informally. Any potential subjects still interested in enrolling will be requested to attend a presentation informing them about the vaccines, entry criteria, study procedures and possible risks of participating. After the presentation there will be the opportunity to ask questions. Those still wishing to participate will undergo informed consent and then proceed to screening.

VI.6. Randomization
For the *in vivo* portion of the study subjects will be assigned unique identification codes (FLU09 followed by a 3-digit number) in order of inclusion and randomized to 1 of 4 groups: separated by 8 weeks ±7 days (2 groups of 8 and 2 groups of 5). Vaccine allocations will be put into consecutively numbered sealed envelopes prior to study commencement. The box containing the envelopes will be kept in a locked cabinet. Once a decision has been taken to enroll a patient, a member of the study team will request the next consecutive randomization number and vaccine allocation. The envelope will be appropriately documented and then opened. Subjects and clinical staff will be aware of study allocation. However all immunology staff not involved in the clinical phase of the study will not know the allocations.

VI.7. Vaccine Regimens
Subjects will be randomized to receive one of the following 4 regimens. For each regimen the two doses of vaccines will be separated by 8 weeks (± 7 days) (Appendix 2).

Arm 1: FluMist® live intranasal vaccine (LAIV) 0.2mL (0.1mL per nostril): 2 doses separated by 8 weeks (±7 days)

Arm 2: Fluzone® influenza virus vaccine (IIV) 0.5mL intramuscularly: 2 doses separated by 8 weeks (±7 days)

Arm 3: FluMist® live intranasal vaccine 0.2mL (0.1mL per nostril) single dose, then Fluzone® influenza virus vaccine 0.5mL intramuscularly single dose 8 weeks (±7 days) later (LAIV then IIV).

Arm 4: Fluzone® influenza virus vaccine 0.5mL intramuscularly single dose, then FluMist® live intranasal vaccine 0.2 mL(1mL per nostril)single dose 8 weeks (± 7 days) later (IIV then LAIV).

VI.8. Packaging, Labeling and Storage of Vaccines

VI.8.1. Live, Attenuated Influenza Vaccine

Chemical name:	N/A
Generic name:	FluMist® Influenza Virus Vaccine Live, Intranasal
Trade name:	FluMist®
Dosage form:	0.2mL pre-filled, single-use nasal spray
Strength:	Each 0.2mL dose contains $10^{6.5\text{-}7.5}$ FFU (fluorescent focus units) of live attenuated influenza virus reassortments of each of the 3 strains for the 2009/2010 season A/South Dakota/6/2007 (H1N1) (an A/Brisbane/59/2007-like), A/Uruguay/716/2007 (H3N2) (an A/Brisbane/10/2007-like), and B/Brisbane/60/2008.
Manufacturer:	MedImmune Vaccines Inc., Gaithersburg, MD 20878
Source:	Donated by MedImmune
Storage:	2-8°C

VI.8.2. Inactivated Influenza Vaccine

Chemical name:	N/A
Generic name:	Influenza Virus Vaccine
Trade name:	Fluzone®
Dosage form:	0.5ml pre-filled syringe
Strength:	Each 0.5ml dose contains 45 micrograms of hemagglutanin in the recommended ratio of 15 micrograms each, representative of the following 3 prototype strains for the 2009/2010 season: A/Brisbane/59/2007, IVR-148 (H1N1), A/Uruguay/716/2007, NYMC X-175C (an A/Brisbane/10/2007-like strain) (H3N2), and B/Brisbane/60/2008.
Manufacturer:	Sanofi Pasteur Inc., Swiftwater, PA 18370
Source:	Thailand purchase
Storage:	2-8°C

VI.9. Duration of Subject Participation

This study will take up to 12 months from enrollment of the first subject to completion of the *in vivo* portion. Each subject will be followed up for approximately 12 weeks following entry into the study (screening, dosing, follow-up). The total study duration will be 5 years.

A subset of previously enrolled subjects may be invited to participate in an additional phase of the study, which will involve consent for use of stored sample and a possible single blood draw if extra sample is required. No follow-up will be required after the day of blood draw

VI.10. Procedures

VI.10.1. *In vivo* Procedures

VI.10.1.1. Informed Consent Process

Potential subjects will have the study explained in detail during a PowerPoint presentation. The informed consent process will take place before any other study procedures and a unique identification number will be assigned. After review the subject will be asked to sign the consent form. Two copies of the consent form should be completed (or the original photocopied) so that the subject can get a copy and the original copy will be kept for the study records. All volunteers will be recorded in a screening log (see Section XIV.2) and those who are subsequently enrolled will be noted with a subject number.

VI.10.1.2. Pre-Study Screening

The pre-study screening visit may be performed anytime within 30 days of planned dosing. The screening examination will consist of:

- Demographic details.
- Medical history to include medical and surgical history, vaccination history and smoking history.
- Physical examination including vital signs (BP, HR, RR, temp), height and weight.
- Blood draw for CBC and HIV test (3ml).

The HIV testing will be done by Department of Retrovirology, AFRIMS as follows:

Test	Screening Test	Confirmatory Test
HIV-1	GS rLAV HIV-1 EIA (RET-GL-115)	GS HIV-1 Western Blot (RET-GL-127)

Pre- and post- HIV test counseling will be given and, if the subject wishes, referral to a specialist recommended by the US Embassy Medical Unit will be made for subjects testing positive. Subjects may choose not to be informed of their HIV test result and in such a case will simply be told that they do not fulfill all the criteria for enrollment. HIV testing may be waived in subjects who have had a negative HIV test result within 2 months of screening.

Subjects should fulfill inclusion criteria outlined in Section VII.1 and not have any of the exclusion criteria listed in Section VII.2

VI.10.1.3. Scheduled Visits

Day 0: The subject will attend for the first dose of vaccine. The study procedures will be reviewed and informed consent re-affirmed verbally. The continued good health of each subject will be confirmed by review of the subject's medical and medication history, medical, surgical and social history, and a brief targeted examination including vital signs (BP, HR, RR and temp). A baseline venous blood sample will be collected (60ml for measurement of cells; 10ml for serum for immunology assay) and a nasal wash for baseline secretory antibody measurements performed. A urine pregnancy test will be performed on females of child-bearing age. If the screening test results (CBC and HIV test) are within normal limits and the subject is not pregnant, the subject will be assigned next available randomization number with details of the vaccine regimen contained within a sealed envelope with that randomization number written on it (see Section VI.6).

The first dose of intranasal FluMist® or intramuscular Fluzone® will then be given and the exact time noted. Following dosing, the clinical staff will inspect the spray container or syringe to confirm that the full dose was delivered. The subject will remain under observation from this time until 30 minutes later to ensure there are no early adverse events. They will then be allowed to return home. Subjects will keep a symptom diary for 14 days following administration of the vaccine.

Day 14 (-2 to +3 days): The subject will attend for symptom diary review and vital signs. 70ml blood will be collected (60ml for measurement of cells and 10ml for serum for immunology assay from a venous sample. A nasal wash will be performed.

Day 28 (-2 to +3 days): All subjects will attend for interim history, and vital signs review. 10ml venous blood will be collected for serum for immunology assay. A nasal wash will be performed.

Day 56 (-7 to +7 days): Subjects in all arms will undergo interim history, vitals signs, urine pregnancy test (for females of child-bearing age).10ml venous blood will be collected (10ml for serum for immunology assay). A nasal wash will be performed. Subjects will receive the second dose of either FluMist® or Fluzone® as determined at randomization on Day 0. As before, following dosing the clinical staff will inspect the spray container or syringe to confirm that the full dose was delivered. The subject will remain under observation from this time until 30 minutes later to ensure there are no early adverse events. They will then be allowed to return home. Subjects will keep a symptom diary for 14 days following administration of the vaccine.

Day 70 (12 to 17 days from 2nd dose): Subjects will attend for symptom diary review and vital signs. 70ml blood will be collected (60ml for measurement of cells and 10ml for serum for immunology assay) from a venous sample. A nasal wash will be performed.

Day 84 (26 to 31 days from 2nd dose): All subjects will attend for review, vital signs and brief physical exam. 10ml venous blood will be collected for serum for immunology assay. A nasal wash will be performed. This is the final study visit.

A flow chart of scheduled visits is included in Appendix 3.

VI.10.1.4. Unscheduled Visits

If the subject returns for an unscheduled visit he/she will be evaluated by a study nurse or study physician. This should include history including symptom diary review if appropriate, VS and physical exam. Additional assessments such as laboratory tests will be performed as indicated clinically. A full record of the visit will be made in the study file.

VI.10.1.5. Summary Table of Volunteer Assessments

Procedure	Day (Week) of Study							
	Screening	0	14 (2)	28 (4)	56 (8)	70 (10)	84 (12)	Unscheduled Visit
History	x	x^	x	x	x	x	x	x
Vital signs	x	x^	x	x	x	x	x	x
Physical exam	x	x^					x	x
CBC and HIV* (3ml)	x							
Urine pregnancy test (females)		x^			x^			
Randomization		x^						
Blood draw:<br>Immunology assay (10ml)<br>Cells (60ml)		 x^<br>x^	 x<br>x	 x	 x^	 x<br>x	 x	
Cumulative blood draw (ml)	3	73	143	153	163	233	243	
Nasal wash		x^	x	x	x^	x	x	
Influenza vaccination		x			x			
Symptom diary review			x			x		x

^ = procedure to be done prior to vaccine administration; * HIV test can be waived if subject has had a negative HIV test within 2 months of screening

VI.10.1.5. Additional Blood Draw

Selected subjects who sign the informed consent Addendum may be asked to undergo one additional blood draw of up to 60ml. A study investigator will assess the subject, including vital signs measurement, prior to venepuncture to ensure that he/she is fit for the procedure. The subject will be observed for 30 minutes after blood draw and then discharged from the study.

VI.10.2. Laboratory Procedures

For serum separation, blood will be collected in SST (Serum Separation Tube with clot activator and gel). For peripheral blood mononuclear cells, blood will be collected in heparinized tube and cells will be separated by histoplaque using Leucosep tubes. Serum samples and peripheral blood mononuclear cells will be frozen in liquid nitrogen until use. Laboratory personnel conducting laboratory procedures described in sections VI.10.2.2 to VI.10.2.7 will not be involved in collecting blood, nor have any direct interaction with study participants. Subject specimens will be collected by clinical research staff, de-identified and sent to the AFRIMS Immunology laboratory or other contracted laboratories.

VI.10.2.1. Influenza viruses

A variety of avian influenza viruses will be used, including H5N1 strains isolated from humans and wild birds and human influenza strains (H1N1 and H3N2).

VI.10.2.2. Detection of influenza-specific IgA and IgG antibodies by ELISA

ELISA plates will be coated with trivalent vaccine Fluzone, H5N1 influenza virus vaccine (Sanofi Pasteur Inc.), swine flu H1N1 2009 vaccine (Sanofi Pasteur Inc.), or recombinant influenza proteins overnight at 4^oC and washed with PBS with 0.05% Tween 20. After blocking with 0.5% boiled casein in PBS with 0.1% Tween 20, serial dilutions of serum and nasal wash samples will be added to the plates and incubated for 2 h. Plates will be washed and incubated for 1 hour with secondary indicator antibodies specific for human IgA or IgG. Plates will be washed and a specific substrate (ABTS) will be used to detect and quantify enzymatic activity through color development; absorbance will be read using a microplate spectrophotometer (405 nm). Antibody titers will be calculated as the dilution factor needed to give an optical density of .5. The assay will be conducted at Immunology BSL2 lab, AFRIMS.

VI.10.2.3. Hemagglutination inhibition assay

To detect antibodies that inhibit binding of influenza virus to cells, serum and nasal wash samples will be treated with receptor-destroying enzyme and subsequently heat-inactivated{Louisirirotchanakul, 2007}. Serial dilutions will be preincubated with pre-determined concentration of inactivated human influenza A, H1N1 (WHO Kit), H3N2 (WHO Kit), swine flu H1N1 2009, and reassortant H5N1 virus in microtiter plates at room temperature for at least 15 min after which red blood cells (RBCs) from goose will be added. RBCs will be allowed to settle at room temperature until cells in the RBC control form a compact negative pattern. If an antigen/antibody reaction occurs, hemagglutination of the RBCs will be inhibited. Inhibition will be graded as "+" for inhibition of HA, "±" for partial inhibition, and "-"for no inhibition. The HI titer is the reciprocal of the last dilution of antiserum that completely inhibits hemagglutination. The HI assay for H1N1, H3N2 will be conducted at Immunology BSL2 Lab, AFRIMS and/or Mahidol University, whereas the HI assay for reassortant H5N1 will be conducted at BIOTEC.

VI.10.2.4. Microneutralization assay

To detect antibodies that inhibit infection of cells with influenza virus, microneutralization assays will be performed using Madin-Darby canine kidney (MDCK) cells. Serum and nasal wash samples will be heat-inactivated (30 min at 56^oC) and serial dilutions will be pre-incubated with H5N1 virus or swine flu H1N1 2009 in 96 well plates. After 1-2 h incubation at 37^oC in a 5% CO2, 100 µl of MDCK cells at 1.5 x 105 /ml will be added to each well and the plates will be incubated for 18 hours. MDCK monolayers will be washed with PBS and fixed in cold 80% acetone for 10 min. The presence of viral protein will be detected by ELISA with a monoclonal antibody to the influenza A NP. The second antibody conjugated with peroxidase will be added and incubated for another 1 h. Plates will be washed, and specific enzyme substrate will be added. The reaction will be stopped with 1 N sulfuric acid. The absorbance will be measured at 490 nm. The average A490 will be determined for quadruplicate wells of virus-infected (VC) and –uninfected (CC) control wells, and a neutralizing endpoint will be determined by using a 50% specific signal calculation. The endpoint titer will be expressed as the reciprocal of the highest dilution of

serum with A490 value less than X, where X = [(average A490 of VC wells) - (average A490 of CC wells)]/2 + (average A490 of CC wells). Sera which tested negative at a dilution of 1/20 will be assigned a titer of 10. The assay will be performed in the BSL3 lab, Mahidol University.

VI.10.2.5. Neuraminidase inhibition assay

Serum and nasal wash samples will be assayed for neuraminidase-specific antibodies by a standard neuraminidase inhibition method {Lambre, 1990} by colorimetric analysis of sialic acid release from fetuin substrate. The assay will be conducted using reassortant H1N1 produced by reverse genetics at BIOTEC. Seven proteins (HA, PB1, PB2, PA, NP, NS, M) of this virus are from A/PR/8/34 (H1N1) and one protein neuraminidase is from A/Vietnam/DT-036/2005 (H5N1). ELISA plates will be coated with fetuin over night at 4^{0}C. The plate will then washed with PBS + 0.5 % Tween 20 and stored at 4^{0}C. The reassortant H1N1 (pre-determined dose that will give optical density of 2.0) will be mixed with serially diluted sera samples (1:1 ratio) for 5 min, added into fetuin coated wells (conducted in biological safety cabinet), and incubated at 37 ^{0}C for 1 h. Controls include virus diluted in PBS-BSA and PBS-BSA without virus. The plate will be washed 6 times in PBS + 0.5 % Tween 20. 100 µl pf peroxidase-labelled peanut agglutinin (1:500) will be added and incubated for another 1 h. Plates will be washed 6 times and 100 µl of peroxidase substart (TMB) added to each well. The reaction will be stopped by adding 50µl of 0.3 M phosphoric acid. Plates will be read in a microplate reader at 450 nm. The antibody titer will be assessed as the highest sample dilution that will give optical density of 1.0 (half of the virus control). Assessment of anti-N1 (seasonal H1N1, reassortant H1N1, and swine flu H1N1 2009) and anti-N2 (seasonal H3N2) antibody levels by neuraminidase inhibition assay will be performed in the BSL2 labs at BIOTEC and/or AFRIMS.

Per AFRIMS policy, receipt of the reassortant virus at AFRIMS will require review by AFRIMS Biosafety Committee and AFRIMS Commander approval before reassortant virus can be received.

VI.10.2.6. Plaque reduction assay

Confluent monolayer of MDCK in 6 or 12 plates will be infected with reassoratant H5N1 or swine flu H1N1 2009 for 1 h. After infection, virus solution will be washed out with PBS and then overlaid with 0.4% agarose containing media, 5%FBS, trypsin and serial diluted sera or nasal washed samples. The plates will be incubated for 3 day and then will be fixed in 10% formalin in PBS and then stained with 0.8 % crystal violet in 50% ethanol. The plaques will be counted. The antibody titer will be assessed as the highest sample dilution that will give a reduction of plaque count of 50%. The assay will be conducted at Mahidol University and/or BIOTEC.

VI.10.2.7. Influenza A (H5) pseudotyped lentiviral particle (H5pp)-based serological assay

Two-fold serial dilutions of heat inactivated serum will be mixed with an equal volume of H5pp in culture medium corresponding to the dose that will generate 10^{5} RLU, incubated for 2 hours at 37°C and then added to a preformed monolayer of MDCK {Garcia, 2010}. After 48 h incubation, luminescence will be read after addition of luciferase substrate. The

neutralization of infection will be detected by measuring the reduction of end-point signal versus control which has no serum on virus. This assay can be conducted in BSL2 lab.

VI.10.2.8. Cytokine production by influenza-specific T-cells

T cell responses will be assessed by intracellular cytokine staining (ICS). Peripheral blood mononuclear cells (PBMC) will be isolated from blood using density gradient centrifugation as described {Thitithanyanont, 2007}. PBMC (10^6 cells) in 200ul will be cultured in 96 wells plates with stimulatory antigens (peptide pool or viral proteins from H5N1 virus, human seasonal influenza virus, or swine flu H1N1 2009). All peptide-or protein-stimulated PBMC cultures will contain 1 ug/ml of anti-CD28 and 1 ug/ml of anti-CD49. Staphylococcal enterotoxin B and media control will be used as positive and negative controls, respectively. After the initial 2 h of stimulation, Golgiplug will be added to inhibit cytokine secretion. After 18-20 h of incubation, cells will be washed and stained for CD4 and CD8. The stained cells will be washed and treated with fixation/permeabilization solution and then stain with antibodies against IFN-g and IL-2. Isotype-match control antibody will be used to confirm the staining specificity. The stained cells will be analyzed by 4-color FACS. Approximately 200,000 to 400,000 events in the lymphocyte gate will be acquired. The samples considered positive will be those in which the percentage of cytokine-staining cells is at least twice that for the background or in which there is a distinct population of brightly cytokine-positive cells. The CD4 T cell population will be analyzed by excluding CD8 T cells, and vice versa for analysis of the CD8 T cell population. The ICS assay will be conducted at Immunology BSL2 lab, AFRIMS.

VI.10.2.9. Antibody staining against matrix 2 protein (M2e) expressed cell line

HEK 293 stably expressing M2e on the cell surface will be stained with a 1: 10 dilution of sera samples and then detected by florescent dye conjugated anti-human antibodies. Stained cells will be analyzed by flow cytometry.

VI.10.2.10. Monoclonal antibody production

Peripheral blood memory B cells from selected subjects will be isolated using a fluorescence-activated cell sorter in a BSL2 laboratory. These isolated B cells will be immortalized with EBV in the presence of TLR 9 ligand CpG ODN. The resulting antibodies produced by the immortalized B cells will be screened for neutralizing antibodies against neuraminidase and hemagglutinin of H5N1 and pandemic H1N1 2009. Positive cultures will then be cloned by limiting dilution.

VI.11. Stopping Rules

There will be no interim analysis and therefore no stopping rules for this study.

VI.12. Accountability Procedures

The Drug Accountability Form will be updated each time a subject receives a dose of vaccine. The form will record the following information for each dose of vaccine: name of vaccine, date administered, subject number and randomization code, number of doses of vaccine at start of study, number of doses of vaccine remaining, signature.

VI.13. Maintenance of Randomization Codes and Procedures for Breaking Codes

This is an open-label study. However all samples for analysis will be coded and assays completed with laboratory technicians blinded as to the randomization sequence. Therefore it is not anticipated that the randomization code will need to be broken until the end of the study for analysis purposes.

VII. SELECTION AND WITHDRAWAL OF SUBJECTS

VII.1. Subject Inclusion Criteria

1. Aged 18-49 years.
2. Healthy as determined by the PI or designate
3. Willing to give informed consent.
4. Willing to attend follow-up appointments and undergo study procedures.
5. US passport holder residing in Bangkok
6. If military, have a letter of approval for participation from the chain of command (Unit Commander).

VII.2. Subject Exclusion Criteria

1. History of hypersensitivity to eggs, egg proteins, gentamicin, gelatin or arginine.
2. Severe or life-threatening reaction to any previous vaccine.
3. History of chronic respiratory illness, including asthma and sinusitis that in the opinion of the investigator warrants exclusion.
4. History of heart disease that in the opinion of the investigator warrants exclusion
5. History of kidney disease that in the opinion of the investigator warrants exclusion
6. Metabolic disease such as diabetes
7. Immunocompromised as determined by the PI, or a household contact of an immunocompromised individual.
8. History of Guillain-Barre syndrome.
9. Blood disease including sickle cell disease and/or bleeding tendency (by history or based on medical records).
10. Any influenza vaccination within 6 months prior to enrollment, or any other vaccine within 30 days of either dose of influenza vaccine.
11. Pregnant woman or nursing mother or unwilling to use reliable contraception during the study period until the final day of follow-up.
12. Blood donation within the preceding 3 months, or screening hemoglobin value of <12.5 g/dl. (males) or <11.6 g/dL (females).
13. Receipt of blood products including immunoglobulins within the preceding 3 months.
14. Receiving any of the following therapies: aspirin, warfarin, theophylline, phenytoin, aminopyrine, immunosuppressive drugs,
15. Receipt of any antiviral agents within 48 hours of vaccine administration.
16. HIV positive on screening blood tests.
17. Any other condition that in the opinion of the study investigator warrants exclusion from the study.

VII.3. Subject Withdrawal Criteria

VII.3.1. Conditions for Subject Withdrawal

The study investigator will make every reasonable effort to have subjects complete the study as designed. If a subject wishes to leave the study at any time, he/she will be permitted to do so. Every reasonable effort will be made by PI or investigator to complete the final clinical assessment. A subject may be withdrawn from the study in any of the following circumstances:

- Serious or severe adverse events attributable or possibly attributable to FluMist® Fluzone®.
- Major violation of the protocol
- Withdrawal of consent
- At the determination of the investigator if he/she feels it is in the subject's best interest
- Termination of the study by the sponsor

VII.3.2. Data to be Collected from Withdrawn Subjects

If a subject withdraws for non-medical reasons the following procedures will be adopted wherever possible:

- The medical risks of withdrawing from the study will be explained to the subject
- A physical examination will be performed
- The subject will be referred to the medical monitor for evaluation of medical needs if deemed appropriate by the investigator.
- The subject will be asked whether he/she still consents to samples and data to be used for analysis. If consent is not withdrawn then samples and data may still be used.

VII.3.3. Follow-up of Withdrawn Subjects

Withdrawn subjects will continue to be followed up if medically indicated.

VII.3.4. Replacement of Withdrawn Subjects

Withdrawn subjects may be replaced at the discretion of the PI and study team.

VIII. CONCOMITENT MEDICATIONS

Any medication the subject takes during the study period is considered concomitant medication. All concomitant medications will be documented. The use of any other investigational drug or vaccine is prohibited. During the study, concomitant medications, particularly anti-viral drugs are to be avoided as much as is possible without compromising the subject's condition. If, in the opinion of the investigator, systemic anti-viral medication is required within 2 weeks of vaccination with FluMist®, the volunteer will be removed from continuation in the study protocol.

IX. ASSESSMENT OF IMMUNE RESPONSE

IX.1. Immune Response Parameters

The following parameters will be used to assess immune responses:

- IgA and IgG antibodies in serum and in nasal wash fluid that
    - react with a variety of H5N1 strains (from birds and from humans), human influenza virus, and recombinant viral proteins (including conserved proteins)
    - inhibit neuraminidase activity of a variety of H5N1 strains and human influenza viruses
    - block infection of cells by a variety of H5N1 strains and human influenza viruses
- T-cell responses that react to protein and/or peptides derived from a variety influenza viruses, including H5N1 strains

IX.2. Methods for Assessing the Immune Response

Serum and nasal washes will be collected for quantification of H5N1 and human influenza - specific antibodies, by ELISA, hemagglutination inhibition, microneutralization, and neuraminidase inhibition assay. Blood will be collected for isolation of peripheral blood mononuclear cells (PBMC) to determine human influenza- and H5N1-specific T-cell responses. Laboratory procedures are described in Section VI.10.2

X. BIOSURETY CONSIDERATIONS

All assays will be conducted at laboratories in Bangkok. Institutional regulations (AFRIMS, Mahidol University, BIOTEC) regarding laboratory safety that are applicable at the time of study execution will be adhered to. Details of which laboratories will be used for individual immunological assays are given in Section VI.10 Laboratory Procedures.

XI. SAFETY

XI.1. Adverse Events

An adverse event (AE) is defined as any reaction, side effect, or untoward event that occurs during the course of the clinical trial, whether or not it was expected or clinically significant, related or unrelated to vaccination**.** For this study, AEs will include events reported by the subject, as well as clinically significant abnormal findings on physical examination or laboratory evaluation. A new illness, symptom, sign or clinically significant clinical laboratory abnormality or worsening of a pre-existing condition or abnormality is considered an AE. Stable chronic conditions, such as arthritis, which are present prior to clinical trial entry and do not worsen are not considered AEs.

All AEs occurring during the course of the clinical trial, defined as from the moment of first phlebotomy until study completion, will be collected, documented, and graded by the PI or other investigators. Symptoms present at enrollment will not be classed as AEs, but any new symptoms or signs occurring after this time would constitute adverse events. Specifically vaccine-emergent adverse events occurring during the first 14 days after vaccination will be

collected using a symptom diary. In addition the CRC or designate will maintain telephone contact with the subjects during the 14 days after each vaccination.

XI.2. Serious Adverse Events

The International Conference on Harmonization (ICH) Guideline for Industry: Clinical Safety Data Management: Definitions and Standards for Expedited Reporting, ICH-E2A March 1995 defines serious adverse event (SAE) or reaction as any untoward medical occurrence that:

- results in death;
- is life-threatening; *(NOTE: The term "life-threatening" in the definition of "serious" refers to an event in which the patient was at risk of death at the time of the event; it does not refer to an event which hypothetically might have caused death if it were more severe.)*
- requires inpatient hospitalization or prolongation of existing hospitalization;
- results in persistent or significant disability/incapacity; or
- is a congenital anomaly/birth defect.
- is an important medical event that, while none of the above, jeopardizes the subject and may require medical or surgical intervention to prevent one of the outcomes listed above. Examples include allergic bronchospasm requiring intensive treatment in an emergency room or at home, blood dyscrasias or convulsions that do not result in hospitalization, or the development of drug dependency or drug abuse.

An unexpected or unanticipated event involving risks to subjects or others is one that is not described as a risk with respect to nature, severity, or frequency in the protocol and/or informed consent form.

XI.3. Detection of AEs and SAEs

At each visit/assessment, the investigator or research nurse will evaluate AEs. AEs should be solicited until the final study assessment, with particular emphasis on those occurring in the first 14 days after each vaccination. A symptom diary will be given to each participant to complete for 14 days following each dose administration. This will form the basis for AE detection. AEs not previously documented in the study will be recorded in the AE report forms. The nature of each experience, date and time (where appropriate) of onset, outcome, course (i.e. intermittent or constant), maximum intensity, action taken with respect to the study and relationship to any study vaccine should be established.

When an AE or SAE occurs, it is the responsibility of the study investigator to review all documentation relative to the event. The investigator will then record all relevant information regarding an AE/SAE on the Adverse Event form. However, there may be instances when copies of medical records for certain cases are appropriate. In this instance, all subject identifiers will be blinded on the copies of the medical records prior to including the information in the medical record.

XI.4. Evaluation of AEs

The study investigator will attempt to establish a diagnosis of the event based on signs, symptoms, and/or other clinical information. In such cases, the diagnosis should be documented as the AE/SAE and not the individual signs/symptoms. Each adverse event must also be described by its duration (start date, time and duration), an assessment of its cause

(vaccine, coexisting disease, concomitant medication, or others), its relationship to vaccine (not related, unlikely, possibly, probably, definitely), and whether it required specific therapy.

The investigator will also make an assessment of severity for each AE reported during the study:

- Mild: Self-limiting or minor symptoms that do not affect activities of daily living and do not require treatment.
- Moderate: Symptoms that require treatment in order to carry out activities of daily living.
- Severe: Symptoms preventing activities of daily living and requiring out-patient treatment.

The assessment will be based on the investigator's clinical judgment. An AE that is assessed as severe should not be confused with a serious adverse event (SAE).

XI.5. Follow up of AEs and SAEs
All AEs regardless of severity will be followed by study investigators until satisfactory resolution. The investigator will ensure that follow-up includes any supplemental investigations as may be indicated to elucidate the nature and/or causality of the AE or SAE. This may include additional laboratory tests or investigations, histopathological examinations, or consultation with other health care professionals.

If a study subject withdraws from the study or if an investigator decides to discontinue the subject from the study because of a SAE, the subject must have appropriate follow-up medical monitoring. Monitoring will continue in order to determine whether the problem prompting hospitalization has resolved or stabilized with no further change expected, or is discovered to be clearly unrelated to study drug, or progresses to death. The Investigator/clinical staff will report the follow-up for serious adverse events as noted above.

XI.6. Post-study AEs and SAEs
A post-study AE/SAE is defined as any event that occurs outside of the AE/SAE detection period (ie. after the final Day 84 assessment). Investigators are not obligated to actively seek AEs or SAEs in former study participants. However, if the investigator learns of any SAE, including a death, at any time after a subject has been discharged from the study, and he/she considers the event reasonably related to the study, the investigator will promptly notify any IRB.

XI.7. Reporting of SAEs and Unanticipated Problems
All serious adverse events will be recorded on the appropriate serious adverse event case report form, followed through resolution by a study physician, and reviewed by a study physician. All unanticipated problems involving risk to subjects or others, serious adverse events related to participation in the study and subject deaths related to participation in the study should be promptly reported by phone, fax, or email to the following:

Division of Human Subjects Protection (DHSP)

Walter Reed Army Institute of Research (WRAIR)
(301) 319-9940 (Tel)
(301) 319 -9163 (Fax)
WRAIRDHSP@amedd.army.mil

A complete written report will follow the initial notification. In addition to the methods above, the complete report will be sent to the Walter Reed Army Institute of Research Division of Human Subjects Protection, 503 Robert Grant Avenue, Silver Spring, MD 20910 and to the U.S. Army Medical Research and Materiel Command, ATTN: MCMR-ZB-PH, 504 Scott Street, Fort Detrick, Maryland 21702-5012.

The medical monitor is required to review all unanticipated problems involving risk to subjects or others, serious adverse events and all subject deaths associated with the protocol and provide an unbiased written report of the event. At a minimum, the medical monitor must comment on the outcomes of the event or problem and in case of a serious adverse event or death, comment on the relationship to participation in the study. The medical monitor must also indicate whether he/she concurs with the details of the report provided by the PI. Reports for events determined by either the investigator or medical monitor to be possibly or definitely related to participation and reports of events resulting in death must be promptly forwarded to the WRAIR IRB. The WRAIR DHSP will in turn be responsible for reporting to USAMRMC ORP HRPO as stipulated in SOP No. UWZ-C-636 (17 November 2008).

XI.8. Monitoring

A Medical Monitor will be appointed for the study. The Medical Monitor will be a qualified physician, who is not associated with the protocol, who is able to provide medical care to research subjects for conditions that may arise during the conduct of the study, and who is able to monitor subjects during the conduct of the study. The Medical Monitor should perform visits approximately every 4 weeks until close of study. As mentioned in Section X.7 the Medical Monitor must review all unanticipated problems, SAEs and deaths associated with the protocol.

In addition there will be an in-house study monitor assigned to this study to ensure compliance with GCP/ICH standards. Monitoring will consist of a pre-study site visit, regular monitoring visits and a post-study visit.

XII. STATISTICAL METHODS

XII.1. Sample Size and Power

This is a descriptive study designed to detect trends in safety and immune responses that would warrant expanded investigations of potentially promising combinations {Lancaster, 2004} {Browne, 1995}. The sample size of 8 subjects in arms 3 and 4 and 5 in arms 1 and 2 is in the range of what is considered adequate for phase I trails and is not based on sample size and power calculations{Stephenson, 2005}{Lin, 2006}{Nicholson, 2001}.

Recruitment of subjects is likely to be affected by the limited number of US citizens resident in Bangkok who fulfill the eligibility criteria. Hence there will be no stratification of enrolment based on gender; this and other possible confounders (such as prior receipt of influenza vaccines) can be addressed in future large-scale trials.

XII.2. Study Outcome Analysis

Primary Analysis
Immunological data and other continuous variables will be expressed as geometric means and standard deviations if the data are normally distributed, and the vaccine groups compared using parametric tests. If not normally distributed they will be expressed as medians and the range and interquartile range given; comparison between groups will then be by non-parametric tests.

Secondary Analyses
In vivo (AE) data will be analyzed as follows: categorical data will be presented using counts and percentages, whilst continuous variables will be presented using the mean, (standard deviation) or median (range and interquartile range), and number of subjects. Percentages will be rounded to a maximum of one decimal place. All statistical tests will be performed using a 5% significance level, leading to 95% confidence intervals. Differences in incidence of reported AEs between doses and vaccines will be determined. Statistical analyses will be carried out in-house using Stata 10.0 (College Station, Texas).

XIII. ETHICAL CONSIDERATIONS

XIII.1. Study Approval

Scientific approval for this study will be obtained from the Scientific Review Committee at AFRIMS. Ethical approval will be sought from the WRAIR IRB (FWA 00000015) and the USAMRMC ORP HRPO. This study will be governed by ICH GCP guidelines.

XIII.2. Risks and Benefits of Participating

Benefits

The study will benefit participating subjects in that they will receive a currently licensed influenza vaccine of proven efficacy. IIVs such as Fluzone have a vaccine efficacy of 70-90% in healthy adults if the vaccine strains are well matched to the circulating influenza strains (Fiore 2007). If the strains are not well matched then efficacy will be lower. In healthy adults there is a theoretical benefit of enhanced protection against vaccine strains following the second dose though (unlike children) this has not been rigorously evaluated in clinical trials.

Risks

The main risks to individual subjects are as follows:

- Phlebotomy. Discomfort and pain at venipuncture sites are possible. Subjects will be counseled to return to the clinic if local infection is suspected.

- HIV testing. A positive HIV test result may cause emotional distress. Pre- and post-test counseling will be given and referral to a specialist recommended by the US Embassy Medical Unit made if the subject wishes. Subjects may choose not to be informed of their HIV test result and in such a case will simply be told that they do not fulfill all the criteria for enrollment.
- Nasal wash. Mild nasal and/or sinus discomfort is possible during the procedure but should resolve as soon as the procedure is finished.
- Allergic reaction to FluMist® or Fluzone®. A history of prior sensitivity to any of the components of the study vaccines will have been sought at pre-study screening and those subjects excluded. From FluMist® post-licensure reports the rate of allergic reaction following single-dose administration is very low (2 per million doses). For Fluzone® the risk of allergic reaction is described in the package insert as rare and when it occurs it is presumably related to residual egg proteins or to antibiotics used in the manufacturing process. Following 2 doses of either vaccine it is possible there may be a greater chance of allergic reaction but the exact risk is unknown.
- Mild respiratory symptoms. These are known to occur more frequently in the 10 days after vaccination in FluMist® recipients (first dose only) than following placebo (approximately 15% more common). Vaccination with IIV is not associated with occurrence of respiratory symptoms.
- Local reactions. Recipients of Fluzone® may experience soreness at the vaccination site (affects 10-64% of subjects) which can persist for 1-2 days. After a second dose of IIV one study in children aged 5-8 years found more subjects experienced soreness after the 2nd dose given 4 weeks later than after the 1st dose (71 and 55% respectively). This has not been noted in other studies of dual dosing in children or in adults with chronic medical conditions.
- Secondary transmission of FluMist® vaccine virus to a close contact (less than 1%). Because of acquired immunity to the vaccine viruses recipients of the 2-dose regimen should not be at any increased risk of secondary transmission following the 2nd dose.
- Neurological risks. There are no known neurological risks with FluMist® vaccination. With IIV vaccination neurological events have been reported rarely. The most important is Guillain-Barre syndrome (risk just over 1 additional case per 1 million vaccinated with single dose). Other rare but reported neurological events have been associated temporally with IIV, including encephalopathy, optic neuritis, partial facial paralysis and brachial plexus neuropathy. These are almost always self-limiting.

These risks will be discussed at the time of study enrollment and in the informed consent document.

XIII.3. Blood Draws

The total volume of blood drawn in this study will be approximately 243 ml over 12 weeks. This is much lower than the generally accepted maximum amount of 550 ml over a 3-month period. The maximum draw on any day will be on Days 0, 14 and 70 when 70ml will be drawn. The minimum period between blood draws is 14 days.

An additional 60ml may be requested from selected subjects after the original follow-up period has been completed, and at least 12 months after completion of the subjects' original study activities.

XIII.4. Sample Storage and Donation for Future Use

During the trial specimens will be stored in a safe place at the study site. They will be regularly transferred to AFRIMS where they may be stored for up to 20 years. No one will have access to the specimens without the explicit permission of the PIs. Samples that are unstable may be disposed of after consultation with the Sponsor. After the study is completed residual specimens will be retained and only used for purposes mentioned in the consent form. Specimens that are sent to other labs in Bangkok (as listed in Section VI.10.2) for analysis will be returned to AFRIMS for storage, as detailed above, once the analysis is complete.

XIV. DATA HANDLING AND RECORD KEEPING

XIV.1. Recording of Data

Subject clinical data will be recorded by the investigator or study nurse using the paper CRFs and entered into an electronic database. Laboratory data will be recorded electronically in the Department of Immunology and uploaded to the study electronic database. All data will be retained according to ICH guidelines. After 5 years the Sponsor will be consulted regarding its disposition or continued storage of raw data.

XIV.2. Confidentiality and Access to Data

All data and medical information obtained about screened study volunteers will be considered privileged and held in confidence. Individual subjects will not be identified on any laboratory samples, CRFs or in any presentation of study results. Instead, subjects enrolling in the study will be issued a unique identification code, which will be used on all study files and clinical sample labels. The key to the code and documents containing personal information, including the screening log, will be kept in a locked cabinet at the US Embassy Clinic during study execution only or at AFRIMS with access restricted to named study personnel (PI, CRC and clinical investigators). All personal study subject data collected and processed for the purposes of this study should be managed by the investigators and his/her staff with adequate precautions to ensure the confidentiality of those data, and in accordance with applicable national and/or local laws and regulations on personal data protection.

Monitors, auditors and other authorized agents, the United States Army Medical Research and Materiel Command, and the ethics committees approving this research will be granted direct access to the study subjects' original medical records for verification of clinical trial procedures and/or data, without violating the confidentiality of the subjects, to the extent permitted by the law and regulations. In any presentations of the results of this study at meetings or in publications, the subjects' identity will remain confidential.

Subject names will also be added to the Volunteer Registry Database as required by the US Army Medical Research and Materiel Command (USAMRMC) whenever human

volunteers are used in research studies. This database is maintained only for patient safety and will be kept in a secure location at Fort Detrick. The information to be entered into this confidential database includes name, address, social security number, study name, and dates. The information will be stored for a minimum of 75 years.

XV. QUALITY CONTROL AND QUALITY ASSURANCE

AFRIMS has approved SOPs/SSPs that govern QC/QA that will be followed during the course of this protocol.

XVI. COMPENSATION

Compensation will be provided throughout the study, and subjects will receive partial compensation if they leave the study before completion. 25% of compensation for scheduled visits will be paid at the final visit to encourage participants not to drop out from the study early. The estimated compensation for completion of the trial will be approximately $300; $30 for screening, $45 per study-related visit, and $15 per unscheduled visit. This compensation takes into consideration lost earnings, food, traveling expenses to and from the study site and discomfort from phlebotomy. Volunteers who are Active Duty military will receive compensation in line with current guidelines (24USC30), but should not exceed compensation paid to non-military volunteers.

Emergency Medical Care: If a subject needs emergency medical care while at the research site, he/she is entitled to receive such care at the research site and/or elsewhere if necessary. If the injury or illness requiring emergency care was caused by the research, any medical care provided by AFRIMS will be at no cost. If the injury or illness was not caused by the research, the subject or his/her insurance may be billed by AFRIMS for such care. Medical care provided by the US Embassy Medical Unit will be at no cost if the subject is a DoD or State Department employee. If not a DoD or State Department employee, the subject or his/her insurance may be billed for medical care provided by the US Embassy Medical Unit.

Non-Emergency Care: Any subject who believes he/she has an injury or illness caused by the research should inform the Principal Investigator, Dr. Delia Bethell.

If the subject is an eligible DoD or State Department employee, he/she may receive medical care at the US Embassy Medical Unit free of charge. The Army will not pay for transportation to and from the clinic. If appropriate medical care is not available at the US Embassy Medical Unit, or the subject is not eligible for care at the US Embassy Medical Unit, he/she will need to seek medical care elsewhere in Bangkok. The subject or his/her insurance company will be responsible for cost of this medical care. The insurance company may not cover some or all expenses for research-related injury, so potential subjects will be advised to check with their insurance companies to determine coverage.

Out of pocket expenses: If out of pocket for medical care are paid for injuries caused by this research study, the subject may contact the Principal Investigator to request reimbursement. Reimbursement cannot be guaranteed, however. The Principal Investigator will coordinate with the US Army Medical Research and Materiel Command (USAMRMC) Office of the Staff Judge Advocate (legal office), in Fort Detrick, Frederick, Maryland, at (301) 619-7663/2221 to process a request for reimbursement. Subjects may also contact the legal office directly.

XVII. FINANCING AND INSURANCE

An application for funding for this trial has been submitted to the MedImmune Investigator Initiated Research Program.

XVIII. ROLES AND RESPONSIBILITIES

Dr. Delia Bethell, Principal Investigator: protocol and consent form design, execution and supervision of all clinical work. Responsible for all *in vivo* aspects of the study to include: supervision and monitoring of research staff, protocol compliance and QA/QC plan execution, and completion of AE documentation, clinical aspects of the study and the final study report and publication. Reports SAEs to IRBs, RCQ and USAMMDA. Performs all duties in accordance with GCP Guidelines.

Dr. David Saunders and Dr Stephen Thomas, Clinical Investigators: protocol and consent form design, execution and supervision of all clinical work. Responsible for all clinical aspects of study to include: supervision and monitoring of research staff, protocol compliance and QA/QC plan execution, and completion of AE documentation and the clinical sections of the final study report and publication. Report SAEs to IRBs, RCQ and USAMMDA. Perform all duties in accordance with CITI Guidelines.

Dr. Sathit Pichyangkul, and Dr Stuart Tyner, Laboratory Investigators: protocol design, supervision and monitoring of *in vitro* portion of study, *in vitro* portion of the final study report and publication. Perform all duties in accordance with CITI Guidelines.

Dr. Mark Fukuda, Consultant: protocol and consent form design, writing of final study report.

Ms. Nichapat Uthaimongkol, Clinical Research Coordinator (CRC): responsible for attending team meetings regarding *in vivo* portion of the research protocol; responsible for ensuring enrolled subjects meet eligibility criteria; ensuring informed consent documents have been signed and witnessed; responsible for protocol compliance, that all AEs have been documented and reported, that follow-up appointments are kept, reporting data trends to the PI, establishing practices to ensure the quality of the data, and ensuring conduct of trial follows GCP Guidelines. Responsible for supervising other study nursing staff, overseeing vaccine administration and blood draws and arranging volunteer follow-up schedules.

Mr. Kosol Yongvanitchit, Laboratory Coordinator: responsible for attending team meetings regarding research laboratory procedures; responsible for ensuring any *in vitro* procedure compliance with protocol, SOPs, and SSPs. He will not be aware of subject randomization.

XIX. CONDITIONS FOR MODIFYING OR TERMINATING THE PROTOCOL

This study will be conducted in accordance with this protocol unless modified in writing. The protocol will not be modified or abandoned without consultation with the sponsor and notification of the principal investigators, subject to the terms outlined below.

- **Protocol Amendment:** A written description of a change(s) to or formal clarification of a protocol. This includes the addition of investigators.

- **Protocol violations** occur when: there is non-adherence to the protocol that results in a significant added risk to the study subject; or, the study subject or investigator has failed to adhere to significant protocol requirements; however, the study subject is enrolled and/or continuing in the study without prior sponsor approval; or there is non-adherence to GCP.

- **Protocol deviations** occur when: there is non-adherence to study procedures or schedules, as specified by the protocol, which do not involve inclusion/exclusion or primary endpoint criteria. The significance of the deviation is based on the frequency of the deviation and/or impact on study objectives. Deviations include: events that are outside of the control of the investigator (e.g. study subject missed visit window) or study parameters that are not part of primary endpoint (e.g. respiratory rate) not performed at a visit.

Protocol deviations will be reported to the WRAIR IRB as part of the annual report/continuing review report. Any violation or deviations to the protocol that may have an effect on the safety or rights of the subject or the integrity of the study must be reported to the WRAIR IRB as soon as the deviation/violation is identified. The DHSP will be responsible for reporting to USAMRMC ORP HRPO.

Ethical Review of Amendments

Major modifications to the research protocol and any modifications that could potentially increase risk to subjects must also be submitted to WRAIR IRB and USAMRMC ORP HRPO for approval prior to implementation. Amendments that do not increase risk to subjects will be submitted to the DHSP for expedited review by WRAIR IRB. Reporting of such amendments to the USAMRMC ORP HRPO will be submitted by the WRAIR DHSP with the continuing reivew report to HRPO for acceptance.

Continuing Review and Protocol Changes

Continuing review should occur during the study execution and also upon study completion. Continuing Review requires the submission of protocol and SOP changes to include: participant population, recruitment methods, research procedures, study instruments, study sites, major research personnel, and conflict of interest issues. These

changes should be approved by the IRBs prior to implementation. A copy of the continuing review report and the local IRB approval notification will be submitted to the WRAIR IRB. After the continuing review report is approved by the WRAIR IRB, the approved CRR will be submitted to the USAMRMC ORP HRPO. The final study report and local IRB approval notification will be submitted to the WRAIR IRB. In accordance with SOP No. UWZ-C-636 protocol lifecycle actions will be reported to the WRAIR IRB alone, and headquarters-level reporting will be the responsibility of the WRAIR DHSP

XX. REFERENCES

Ambrose CS, W. R., Connor EM (2006). "Live attenuated influenza vaccine in children." Semin Pediatr Infect Dis **17**(4): 206-12.

Ashkenazi S, V. A., Aristegui J, Esposito S, McKeith DD, Klemola T, Biolek J, Kuhr J, Bujnowski T, Desgrandchamps D, Cheng SM, Skinner J, Gruber WC, Forrest BD (2006). "Superior relative efficacy of live attenuated influenza vaccine compared with inactivated influenza vaccine in young children with recurrent respiratory tract infections." Pediatr Infect Dis J **25**(10): 870-9.

Beigel, J. H., J. Farrar, et al. (2005). "Avian influenza A (H5N1) infection in humans." N Engl J Med **353**(13): 1374-85.

Belshe R, L. M., Walker RE, Stoddard J, Mendelman PM. (2004). "Safety, immunogenicity and efficacy of intranasal, live attenuated influenza vaccine." Expert Rev Vaccines **3**(6): 643-54.

Belshe RB, E. K., Vesikari T, Black SV, Walker RE, Hultquist M, Kemble G, Connor EM. (2007). "Live attenuated versus inactivated influenza vaccine in infants and young children." N Engl J Med **356**(7): 685-96.

Belshe RB, M. P., Treanor J, King J, Gruber WC, Piedra P, Bernstein DI, Hayden FG, Kotloff K, Zangwill K, Iacuzio D, Wolff M. (1998). "The efficacy of live attenuated, cold-adapted, trivalent, intranasal influenzavirus vaccine in children." N Engl J Med **338**(20): 1405-12.

Belshe RB, N. K., Black SV, Shinefield H, Cordova J, Walker R, Hessel C, Cho I, Mendelman PM. (2004). "Safety, efficacy and effectiveness of live, attenuated, cold-adapted influenza vaccine in an indicated population aged 5-49 years." Clin Infect Dis **39**(7): 920-7.

Beltramello, M., K. L. Williams, et al. (2010). "The human immune response to Dengue virus is dominated by highly cross-reactive antibodies endowed with neutralizing and enhancing activity." Cell Host Microbe **8**(3): 271-283.

Bergen R, B. S., Shinefield H, Lewis E, Ray P, Hansen J, Walker R, Hessel C, Cordova J, Mendelman PM (2004). "Safety of cold-adapted live attenuated influenza vaccine in a large cohort of children and adolescents." Pediatr Infect Dis J **23**(2): 138-44.

Beyer WE, P. A., de Jong JC, Osterhaus AD (2002). "Cold-adapted live influenza vaccine versus inactivated vaccine: systemic vaccine reactions, local and systemic antibody response, and vaccine efficacy. A meta-analysis." Vaccine **20**(9-10): 1340-53.

Bresson JL, P. C., Launay O, Gerdil C, Saville M, Wood J, Hoschler K, Zambon MC (2006). "Safety and immunogenicity of an inactivated split-virion influenza A/Vietnam/1194/2004 (H5N1) vaccine: phase I randomised trial." Lancet **367**(9523): 1657-64.

Browne R.H. (1995). "On the use of a pilot sample for sample size determination." Stat Med **14**(17): 1933-1940.

CDC. (2008). "Influenza virus vaccine 2008-2009 season." from www.fda.gov/cber/flu/flu2008.htm.

Claas, E. C., A. D. Osterhaus, et al. (1998). "Human influenza A H5N1 virus related to a highly pathogenic avian influenza virus." Lancet **351**(9101): 472-7.

Corti, D., J. P. Langedijk, et al. (2010). "Analysis of memory B cell responses and isolation of novel monoclonal antibodies with neutralizing breadth from HIV-1-infected individuals." PLoS One **5**(1): e8805.

Cox RJ, B. K., Ogra P (2004). "Influenza virus: immunity and vaccination strategies. Comparison of the immune response to inactivated and live, attenuated influenza vaccines." Scand J Immunol **59**(1): 1-15.

Doherty, P. C., S. J. Turner, et al. (2006). "Influenza and the challenge for immunology." Nat Immunol **7**(5): 449-55.

Englund JA, W. E., Fairchok MP, Monto AS, Neuzil KM (2005). "A comparison of 2 influenza vaccine schedules in 6- to 23-month-old children." Pediatrics **115**(4): 1039-47.

Ehrlich HJ, M. M., Oh H, Tambyah PA, Joukhadar C, Montomoli E, Fisher D, Berezuk G, Fritsch S, Low-Baselli A, Vartian N, Bobrovsky R, Pavlova BG, Pollabauer EM, Kistner O, Barrett N (2008). "A clinical trial of a whole-virus H5N1 vaccine derived from cell culture." N Engl J Med **358**(24): 2573-84.

Ernst W, A. et al (2008) "Protection against H1, H5, H6 and H9 influenza A infection with liposome matrix 2 epitope vaccines." Vaccine **24**:156-68.

Fauci, A. S. (2006). "Pandemic influenza threat and preparedness." Emerg Infect Dis **12**(1): 73-7.

Fiore AE, S. D., Haber P, Iskander JK, Uyeki TM, Mootrey G, Bresee JS, Cox NJ (2007). "Prevention and control of influenza: Recommendations of the Advisory Committee on Immunization Practices (ACIP), 2007." MMWR **56**(RR06): 1-54.

Fleming D, C. P., Wahn U, Klemola T, Schlesinger Y, Langussis A, Oymar K, Garcia ML, Krygier A, Costa H, Heininger U, Pregaldien JL, Cheng SM, Skinner J, Razmpour A, Saville M, Gruber WC, Forrest B (2006). "Comparison of the efficacy and safety of live attenuated cold-adapted influenza vaccine, trivalent, with trivalent inactivated influenza virus vaccine in children and adolescents with asthma." Pediatr Infect Dis J **25**(10): 860-9.

Garcia JM, Lagarde N, Ma ESK, de Jong MD, Peiris JSM (2010). „Optimization and evaluation of an influenza A (H5) pseudotyped lentiviral particle-based serological assay." J Clin Virol 47:29.

Gomez CE, N, J. L., et al (2007). "Head-to head comparison on the immunogenicity of two HIV/AIDS vaccine candidates based on the attenuated poxvirus strains MVA and NYVAC co-expressing in a single locus the HIV-1BX08 gp120 and HIV-1 (IIIB) gag-Pol-Nef protein of clade B" Vaccine **25**(15):2863-85.

Guerrero IC, R. H., Brandling-Bennett AD, Hinman AR (1979). "No increased meningoencephalitis after influenza vaccine." N Engl J Med **300**(10): 565.

Haber P, D. F., Angulo FJ, Iskander J, Shadomy SV, Weintraub E, Chen RT (2004). "Guillain-Barré syndrome following influenza vaccination." JAMA **292**(20): 478-81.

Huleatt J, W. et al. (2008) "Potent immunogenicity and efficacy of a universal influenza vaccine candidate comprising a recombinant fusion protein linking influenza M2e to the TLR5 ligand flagellin." Vaccine **26**:201-4.

Ichinohe T., Tamura S, Kawaguchi A, Ninomiya A, Imai M, Itamura S, Odagiri T, Tashiro M, Takahashi H, Sawa H, Mitchell WM, Strayer DR, Carter WA, Chiba J, Kurata T, Sata T, Hasegawa H. (2007). "Cross-protection against H5N1 influenza virus infection is afforded by intranasal inoculation with seasonal trivalent influenza vaccine." J Infect Dis **196**(9): 1313-20.

Izurieta HS, H. P., Wise RP, Iskander J, Pratt D, Mink C, Chang S, Braun MM, Ball R (2005). "Adverse events reported following live, cold-adapted, intranasal influenza vaccine." JAMA **294**(21): 2720-5.

Jackson LA, H. S., Mendelman PM, Huggins L, Cho I, Rhorer J (1999). "Safety of a trivalent live attenuated intranasal influenza vaccine, FluMist, administered in addition to parenteral

trivalent inactivated influenza vaccine to seniors with chronic medical conditions." Vaccine **17**(15-16): 1905-9.

Katz JM, Garg S., Sambhara S. Influenza vaccines: current and future strategies. In: Influenza Virology: Current Topics. Ed. Kawaoka Y., Caister Academic Press, Norfolk, England.

Kietel WA, C. R., Atmar RL, Nino D, Cate TR, Couch RB. (2008). "Increasing doses of an inactivated influenza A/H1N1 vaccine induce increasing levels of cross-reacting antibody to subsequent antigenically different variants." J Infect Dis **198**(1 October): 1016-8.

Kietel WA, C. R., Quarles JM, Cate TR, Baxter B, Maassab HF (1993). "Trivalent attenuated cold-adapted influenza virus vaccine: reduced viral shedding and serum antibody responses in susceptible adults." J Infect Dis **167**(2): 305-11.

King JC Jr, F. P., Zangwill KM, Weinberg GA, Wolff M, Yan L, Newman F, Belshe RB, Kovacs A, Deville JG, Jelonek M (2001). "Safety, vaccine virus shedding and immunogenicity of trivalent, cold-adapted, live attenuated influenza vaccine administered to human immunodeficiency virus-infected and noninfected children." Pediatr Infect Dis J **20**(12): 1124-31.

King JC Jr, T. J., Fast PE, Wolff M, Yan L, Iacuzio D, Readmond B, O'Brien D, Mallon K, Highsmith WE, Lambert JS, Belshe RB (2000). "Comparison of the safety, vaccine virus shedding, and immunogenicity of influenza virus vaccine, trivalent, types A and B, live cold-adapted, administered to human immunodeficiency virus (HIV)-infected and non-HIV-infected adults." J Infect Dis **181**(2): 725-8.

Lalor P, A. et al. (2008). “Plasmid DNA-based vaccines protect mice and ferrets against lethal challenge with A/Vietnam/1203/04 (H5N1) influenza virus.” J. Infect Dis 197:1643-52.

Lancaster G.A., Dodd S., Williamson P.R. (2004). “Design and analysis of pilot studies: recommendations for good practice.” J. Eval Clin Prac 10(2):307-312.

Lanzavecchia, A. and F. Sallusto (2009). "Human B cell memory." Curr Opin Immunol **21**(3): 298-304.

Leroux-Roels I, B. A., Vanwolleghem T, Drame M, Clement F, Hons E, Devaster JM, Leroux-Roels G (2007). "Antigen sparing and cross-reactive immunity with an adjuvanted rH5N1 prototype pandemic influenza vaccine: a randomised controlled trial." Lancet **370**(9587): 544-5.

Lin J, Z. J., Dong X, Fang H, Chen J, Su N, Gao Q, Zhang Z, Liu Y, Wang Z, Yang M, Sun R, Li C, Lin S, Ji M, Liu Y, Wang X, Wood J, Feng Z, Wang Y, Yin W (2006). "Safety and immunogenicity of an inactivated adjuvanted whole-virion influenza A (H5N1) vaccine: a phase I randomised controlled trial." Lancet **368**(9540): 991-7.

Louisirirotchanakul S, L. H., Wiriyarat W, Sangsiriwut K, Chaichoune K, Pooruk P, Songserm T, Kitphati R, Sawanpanyalert P, Komoltri C, Auewarakul P, Puthavathana P (2007). "Erythrocyte binding preference of avian influenza H5N1 viruses." J Clin Microbiol **45**(7): 2284-6.

Lu X, E. L., Desheva JA, Nguyen DC, Rekstin A, Stephenson I, Szretter K, Cox NJ, Rudenko LG, Klimov A, Katz JM (2006). "Cross-protective immunity in mice induced by live-attenuated or inactivated vaccines against highly pathogenic influenza A (H5N1) viruses." Vaccine **24**(44-46): 6588-93.

Miotti PG, N. K., Dallabetta GA, Farzadegan H, Margolick J, Clements ML (1989). "The influence of HIV infection on antibody responses to a two-dose regimen of influenza vaccine." JAMA **262**(6): 779-83.

McConkey SJ, R. W. H., et al (2003). "Enhanced T-cell immunogenicity of plasmid DNA vaccines boosted by recombinant modified vaccinia virus Ankara in humans" Nat Med **9**(6):729-35.

Neuzil KM, J. L., Nelson J, Klimov A, Cox N, Bridges CB, Dunn J, DeStefano F, Shay D (2006). "Immunogenicity and reactogenicity of 1 versus 2 doses of trivalent inactivated influenza vaccine in vaccine-naive 5-8-year-old children." J Infect Dis **194**(8): 1032-9.

Nichol KL, M. P., Mallon KP, Jackson LA, Gorse GJ, Belshe RB, Glezen WP, Wittes J (1999). "Effectiveness of live, attenuated intranasal influenza virus vaccine in healthy, working adults: a randomized controlled trial." JAMA **282**(2): 137-44.

Nicholson KG, C. A., Podda A, Stephensen I, Wood J, Ypema E, Zambon MC (2001). "Safety and antigenicity of non-adjuvanted and MF59-adjuvanted influenza A/Duck/Singapore/97 (H5N3) vaccine: a randomised trial of two potential vaccines against H5N1 influenza." Lancet **357**(9272): 1937-43.

Peiris, J. S., W. C. Yu, et al. (2004). "Re-emergence of fatal human influenza A subtype H5N1 disease." Lancet **363**(9409): 617-9.

Pichyangkul, S., M. Gettayacamin, et al (2004). "Pre-clinical evaluation of the malaria vaccine candidate P. Falciparum MSP1 (42) formulated with novel adjuvant or with alum". Vaccine **22** (29-30):3831-40.

Pichyangkul, S., U. Kum-Arb, et al (2008). "Preclinical evaluation of the safety and immunogenicity of a vaccine consisting of Plasmodium falciparum liver-stage antigen 1 with adjuvant AS01B administered alone or concurrently with the RTS,S/AS01B vaccine in rhesus primates" Infect Immun **76**(1): 229-38.

Piedra PA, G. M., Riggs M, Herschler G, Fewlass C, Watts M, Kozinetz C, Hessel C, Glezen WP (2005). "Live attenuated influenza vaccine, trivalent, is safe in healthy children 18 months to 4 years, 5 to 9 years, and 10 to 18 years of age in a community-based, nonrandomized, open-label trial." Pediatrics **116**(3): e397-407.

Piedra PA, Y. L., Kotloff K, Zangwill K, Bernstein DI, King J, Treanor J, Munoz F, Wolff M, Cho I, Mendelman PM, Cordova J, Belshe RB (2002). "Safety of the trivalent, cold-adapted influenza vaccine in preschool-aged children." Pediatrics **110**(4): 662-72.

Puig BJ, G. V. (2007). "MF59-adjuvanted subunit influenza vaccine: an improved interpandemic influenza vaccine for vulnerable populations." Expert Rev Vaccines **6**(5): 659-65.

Redding G, W. R., Hessel C, Virant FS, Ayars GH, Bensch G, Cordova J, Holmes SJ, Mendelman PM (2002). "Safety and tolerability of cold-adapted influenza virus vaccine in children and adolescents with asthma." Pediatr Infect Dis J **21**(1): 44-8.

Reeve P, G. B., Moritz A, Liehl E, Kunz C, Hofmann H, Maassab HF (1980). "Studies in man with cold-recombinant influenza virus (H1N1) live vaccines." J Med Virol **6**(1): 75-83.

Retaillaiu HF, C. A., Storr G, Caesar G, Eddins DL, Hattwick MA (1980). "Illness after influenza vaccination reported through a nationwide surveillance system, 1976-1977." Am J Epidemiol **111**(3): 270-8.

Sandbulte MS, G. S. J., Boon ACM, Smith LR, Trenor JJ, Webby RJ (2007). "Cross-Reactive neuraminidase antibodies afford partial protection against H5N1 in mice and are present in unexposed humans". PLoS Medicine **4**(2) 0265-71.

Schonberger LB, B. D., Sullivan-Bolyai JZ, Keenlyside RA, Ziegler DW, Retailliau HF, Eddins DL, Bryan JA (1979). "Guillain-Barre syndrome following vaccination in the National Influenza Immunization Program, United States, 1976--1977." Am J Epidemiol **110**(2): 105-23.

Simmons, C. P., N. L. Bernasconi, et al. (2007). "Prophylactic and therapeutic efficacy of human monoclonal antibodies against H5N1 influenza." PLoS Med **4**(5): e178.

Song JY, C. H., Ha SH, Kee SY, Jeong HW, Kim WJ (2006). "Active influenza immunization in hemodialysis patients: comparison between single-dose and booster vaccination." Am J Nephrol **26**(2): 206-11.

Stephenson I, B. R., Nicholson KG, Podda A, Wood JM, Zambon MC, Katz JM (2005). "Cross-reactivity to highly pathogenic avian influenza H5N1 viruses after vaccination with nonadjuvanted and MF59-adjuvanted influenza A/Duck/Singapore/97 (H5N3) vaccine: a potential priming strategy." J Infect Dis **191**(8): 1210-5.

Stephenson I, N. K., Colegate A, Podda A, Wood JM, Ypema E, Zambon MC (2003). "Boosting immunity to influenza H5N1 with MF59-adjuvanted H5N3 A/Duck/Singapore/97 vaccine in a primed human population." Vaccine **21**(15): 1687-93.

Subbarao K, L. C. (2007). "H5N1 viruses and vaccines." PLoS Pathog **3**(3): e40.

Talbot TR, C. D., Peters J, Doersam JK, Ikizler MR, Sannella E, Wright PE, Edwards KM (2005). "Duration of virus shedding after trivalent intranasal live attenuated influenza vaccination in adults." Infect Control Hosp Epidemiol **26**(5): 494-500.

Tam, J. (2005). Efficacy and safety of a live, attenuated, trivalent influenza vaccine (CAIV-T) in children living in south-east Asia against community acquired culture-confirmed influenza. 2005 Pediatric Academic Societies Annual Meeting. Washington DC. **57:** 728.

Thitithanyanont A, E. A., Ekchariyawat P, Wiboon-ut S, Limsalakpetch A, Yongvanitchit K, Kum-Arb U, Kanchongkittipohn W, Utaisincharoen P, Sirasinha S, Puthavathana P, Fukuda MM, Pichyangkul S (2007). "High susceptibility of human dendritic cells to avian influenza H5N1 virus infection and protoection byIFN-alpha and TLR ligands." J Immunol **179**(8): 5220-7.

Tanzi E, A. A., Pariani E, Zappa A, Colzani D, Logias F, Perego A, Zanetti A (2007). "Lack of effect of a booster dose of influenza vaccine in hemodialysis patients." J Med Virol **79**(8): 1176-9.

Tompkins SM, Zhao ZA (2007). "Matrix protein 2 vaccine vaccination and protection against influenza viruses, including subtype H5N1". Emerge Infect Dis **13** (3) 426-35.

Traggiai, E., S. Becker, et al. (2004). "An efficient method to make human monoclonal antibodies from memory B cells: potent neutralization of SARS coronavirus." Nat Med **10**(8): 871-875.

Treanor JJ, C., Zangwill KM, Rowe T, Wolff M (2006). "Safety and immunogenicity of an inactivated subvirion influenza A (H5N1) vaccine." N Engl J Med **354**(13): 1343-51.

Treanor JJ, W. B., Masseoud F, Hu-Primmer J, Battaglia R, O'Brien D, Wolff M, Rabinovitch G, Blackwelder W, Katz JM (2001). "Safety and immunogenicity of a recombinant hemagglutinin vaccine for H5 influenza in humans." Vaccine **19**(13-14): 1732-7.

Verrier F, S.B., et al (2000). A human immunodeficiency virus prime-boost immunization regimen in humans induces antibodies that show interclade cross-reactivity and neutralize several X4-R5-, and dualtropic clade B and C primary isolates. J Virol **74**(2): 10025-33.

Vesikari T, K. A., Smith HM, Dunning A, Razmapour A, Saville MK, Gruber WC, Forrest BD (2008). "Safety and tolerability of cold-adapted influenza vaccine, trivalent, in infants younger than 6 months of age." Pediatrics **121**(3): e568-73.

Vesikari T, F. D., Aristegui JF, Vertruyen A, Ashkenazi S, Rappaport R, Skinner J, Saville MK, Gruber WC, Forrest BD (2006). "Safety, efficacy, and effectiveness of cold-adapted

influenza vaccine-trivalent against community-acquired, culture-confirmed influenza in young children attending day care." Pediatrics **118**(6): 2298-312.

Webster RG, P. M., Chen H, Guan Y (2006). "H5N1 outbreaks and enzootic influenza." Emerg Infect Dis **12**(1): 3-8.

Webster RG, Campbell CH (1972). "An inhibition test for identifying the neuraminidase antigen on influenza viruses". Avian Dis **16** (1):1057-66.

World Health Organization (2008) (http://www.who.int/csr/disease/influenza/vaccinerecommendations/en/index.html)

Appendix 1. Flow Diagram of Vaccine Combinations

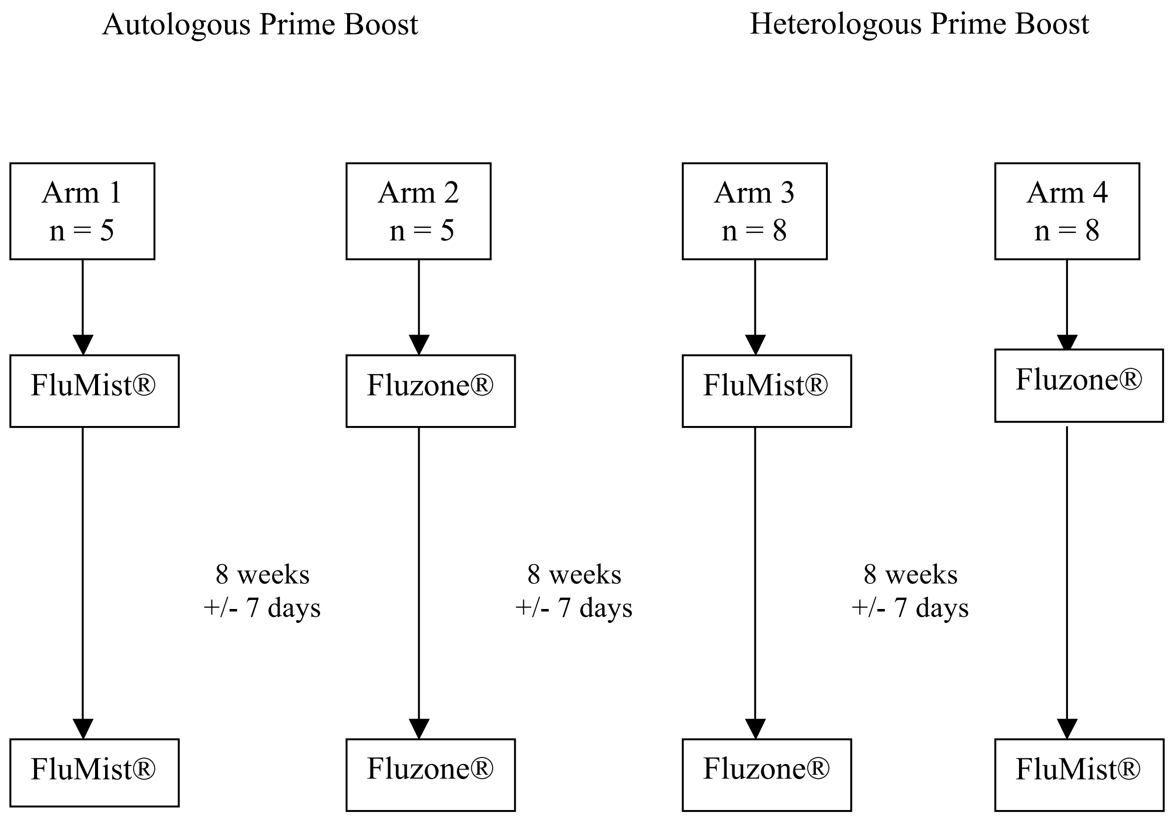

Appendix 2. Flow Chart of Subject Visits

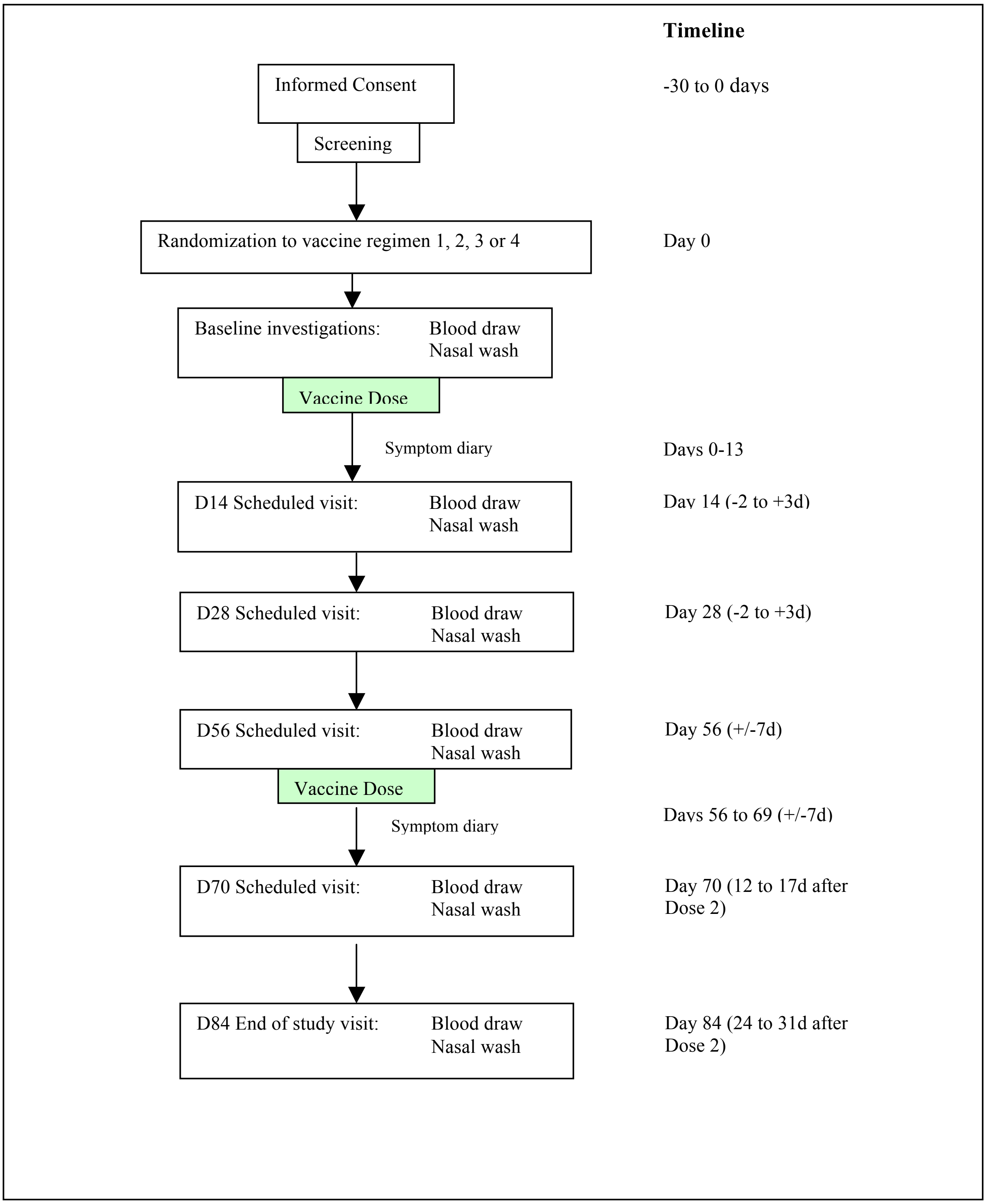

Supplement: Protocol S1 — Study Protocol. (PDF) [file pone.0059674.s004.pdf]
